# Recommendations for connecting molecular sequence and biodiversity research infrastructures through ELIXIR

**DOI:** 10.12688/f1000research.73825.1

**Published:** 2021-12-03

**Authors:** Robert M. Waterhouse, Anne-Françoise Adam-Blondon, Donat Agosti, Petr Baldrian, Bachir Balech, Erwan Corre, Robert P. Davey, Henrik Lantz, Graziano Pesole, Christian Quast, Frank Oliver Glöckner, Niels Raes, Anna Sandionigi, Monica Santamaria, Wouter Addink, Jiri Vohradsky, Amandine Nunes-Jorge, Nils Peder Willassen, Jerry Lanfear

**Affiliations:** 1 Department of Ecology and Evolution and Swiss Institute of Bioinformatics, University of Lausanne, Lausanne, Vaud, 1015, Switzerland; 2 Université Paris Saclay, Versailles, 78026, France; 3 Plazi, Bern, 3007, Switzerland; 4 Institute of Microbiology of the Czech Academy of Sciences, Praha, 142 20, Czech Republic; 5 Institute of Biomembranes, Bioenergetics and Molecular Biotechnologies, CNR, Bari, 70126, Italy; 6 CNRS/Sorbonne Université, Station Biologique de Roscoff, Roscoff, 29680, France; 7 Earlham Institute, Norwich, NR4 7UZ, UK; 8 Department of Medical Biochemistry and Microbiology/NBIS, Uppsala University, Uppsala, Sweden; 9 Department of Biosciences. Biotechnology and Biopharmaceutics, University of Bari “A. Moro”, Bari, 70126, Italy; 10 Life Sciences & Chemistry, Jacobs University Bremen gGmbH, Bremen, Germany; 11 MARUM - Center for Marine Environmental Sciences, University of Bremen, Bremerhaven, 27570, Germany; 12 Alfred Wegener Institute, Helmholtz Center for Polar- and Marine Research, Bremerhaven, 27570, Germany; 13 NLBIF - Netherlands Biodiversity Information Facility, Naturalis Biodiversity Center, Leiden, 2300 RA, The Netherlands; 14 University of Milan Bicocca, Milan, 20127, Italy; 15 DiSSCo - Distributed System of Scientific Collections, Naturalis Biodiversity Center, Leiden, 2300 RA, The Netherlands; 16 Laboratory of Bioinformatics, Institute of Microbiology, Prague, 142 20, Czech Republic; 17 Dept. of Chemistry, UiT The Arctic University of Norway, Tromsø, Norway; 18 ELIXIR Hub, Wellcome Genome Campus, Cambridge, CB10 1SD, UK

**Keywords:** Bioinformatics, Genomics, Sequencing, Data Management, Data Standards, Genetic Resources, Taxonomy

## Abstract

Threats to global biodiversity are increasingly recognised by scientists and the public as a critical challenge. Molecular sequencing technologies offer means to catalogue, explore, and monitor the richness and biogeography of life on Earth. However, exploiting their full potential requires tools that connect biodiversity infrastructures and resources. As a research infrastructure developing services and technical solutions that help integrate and coordinate life science resources across Europe, ELIXIR is a key player. To identify opportunities, highlight priorities, and aid strategic thinking, here we survey approaches by which molecular technologies help inform understanding of biodiversity. We detail example use cases to highlight how DNA sequencing is: resolving taxonomic issues; Increasing knowledge of marine biodiversity; helping understand how agriculture and biodiversity are critically linked; and playing an essential role in ecological studies. Together with examples of national biodiversity programmes, the use cases show where progress is being made but also highlight common challenges and opportunities for future enhancement of underlying technologies and services that connect molecular and wider biodiversity domains. Based on emerging themes, we propose key recommendations to guide future funding for biodiversity research: biodiversity and bioinformatic infrastructures need to collaborate closely and strategically; taxonomic efforts need to be aligned and harmonised across domains; metadata needs to be standardised and common data management approaches widely adopted; current approaches need to be scaled up dramatically to address the anticipated explosion of molecular data; bioinformatics support for biodiversity research needs to be enabled and sustained; training for end users of biodiversity research infrastructures needs to be prioritised; and community initiatives need to be proactive and focused on enabling solutions. For sequencing data to deliver their full potential they must be connected to knowledge: together, molecular sequence data collection initiatives and biodiversity research infrastructures can advance global efforts to prevent further decline of Earth’s biodiversity.

## Introduction

### Sequence data collection initiatives offer opportunities to connect with and feed into biodiversity research infrastructures

Biological diversity represents the full spectrum of the variety of organisms on Earth, at genetic, species, and ecosystem levels, created over millions of years of evolution. Biodiversity is also essential for life itself, for the sustainability of varied communities of interdependent and interacting species at all scales. Anthropocentrically, biodiversity forms the foundation of ecosystem services that are indispensable for human well-being and a healthy planet. Whilst biodiversity is naturally constantly changing, increasingly unsustainable pressures resulting from human activities mean that this variety is currently being lost like never before. Recognising the threat to humanity that this decline poses, governments and international organisations have responded with strategies to protect and restore biodiversity, such as the Intergovernmental Science-Policy Platform on Biodiversity and Ecosystem Services ( IPBES 2021). These and other initiatives also recognise the important roles that genetic and genomic data can play in biodiversity assessment, monitoring, conservation, and restoration, to ensure the long-term health of ecosystem services ( Hoban et al. 2020). This requires infrastructures that make it easier for scientists to exchange knowledge and agree on best practices, as well as to find, share, and connect increasingly large and diverse datasets. As an intergovernmental organisation that develops services and technical solutions to integrate and coordinate life science resources from across Europe, ELIXIR recognises that connecting molecular sequence data with biodiversity research infrastructures will be critical to support global efforts to prevent further declines of biodiversity.

*Biodiversity sequencing and research infrastructure initiatives*

One of the aims of biodiversity research infrastructures is to compile and maintain comprehensive lists of all known species of organisms including their spatio-temporal distributions on Earth, normally within a taxonomic framework and usually with additional associated metadata. Prominent examples that bring together information from multiple sources include the Catalogue of Life (CoL) ( Roskov et al. 2020), the Global Biodiversity Information Facility ( GBIF 2021), the Environmental Research Infrastructures Community ( ENVRI 2021), the Ocean Biodiversity Information System ( OBIS 2021), the Encyclopedia of Life ( Parr et al. 2014), and the Distributed System of Scientific Collections ( DiSSCo 2021). For example, GBIF aims to map diversity in space and time based on natural science collection records, sequence data, biodiversity surveys, human and machine observations, and species lists. The taxonomic frameworks are built from sources of published records such as the Biodiversity Heritage Library and the Biodiversity Literature Repository (BLR), with ongoing efforts to standardise data and make them machine readable and citable ( Agosti & Egloff 2009; Penev et al. 2012; Bénichou et al. 2019). Biodiversity research infrastructures also encompass biobanks (genebanks or seed banks) for conserving genetic resources, of major crops and their wild relatives e.g. collated by Genesys ( Genesys 2021), of livestock breeds managed by the Domestic Animal Diversity Information System ( DAD-IS 2021), or of microbes in the context of food and agriculture or health e.g. managed by the Microbial Resource Research Infrastructure ( MIRRI 2021).

Molecular data collection initiatives are equally as varied, aiming to collate growing amounts of DNA and RNA sequence data, often also employing a taxonomic framework and collecting sample metadata. Notable examples include the principally archival International Nucleotide Sequence Database Collaboration (INSDC) ( Arita et al. 2021) comprising the DNA DataBank of Japan (DDBJ), the European Nucleotide Archive (ENA), and the United States National Center for Biotechnology Information (NCBI) GenBank, as well as the China National GeneBank DataBase ( Wang et al. 2019). More specialised initiatives focus on e.g. ribosomal RNA collections ( Glöckner et al. 2017; Santamaria et al. 2018; Nilsson et al. 2019), microbiome resources ( Mitchell et al. 2020), or metagenomics sequence data ( Meyer et al. 2019).

These examples help to formulate more formal definitions: (i) molecular sequence data collection initiatives are producing and collating reference catalogues of genetic and genomic biodiversity on Earth; and (ii) biodiversity research infrastructures are capturing knowledge from scientific collections, observations, and the literature, and building resources of biodiversity information for all Earth’s organisms. Here we identify opportunities to connect these biodiversity sequence collection initiatives and research infrastructures in a standardised and scalable manner that will greatly enhance the utility of both by facilitating data-to-knowledge research.

*Expanding collections of molecular sequence data*

New technologies and falling sequencing costs are greatly improving the diversity of species sampling through the acquisition of increasing amounts of molecular data. This has led to a growing number of large-scale sequencing data generation initiatives with increasingly ambitious sampling aims covering eukaryotes, prokaryotes, and viruses ( Table 1). For example, the Earth BioGenome Project ( EBP 2021) aims to coordinate the sequencing and characterisation of the genomes of all eukaryotic life, with a vision of creating a new foundation for biology that will deliver solutions for understanding ecosystems, protecting biodiversity, and benefiting human welfare ( Lewin et al. 2018). This involves developing and agreeing on standards for all protocols from specimen collection and identification through to sequencing, assembly, annotation, and analysis. Initiatives are typically geographically or taxonomically focused, such as the Darwin Tree of Life ( DToL 2021) project in Britain and Ireland, The European Reference Genome Atlas initiative ( ERGA 2021), the Vertebrate Genomes Project (VGP) ( Rhie et al. 2020), the i5k Arthropod Genomes Initiative ( i5K Consortium 2013), the 10KP Plant Genomes Project ( Cheng et al. 2018), and others ( Table 1).

**Table 1.** Examples of major molecular sequence data generation and coordination initiatives. A non-exhaustive list of active international projects and umbrella initiatives covering many species and producing (meta) genomes, (meta) transcriptomes, and/or DNA barcodes, with public data deposition.

Initiative name/acronym	Main focus of the initiative	URL/website for further information
1KITE 1’000 Insect Transcriptome Evolution	Transcriptomes, insects	https://www.1kite.org/
1KP 1’000 Plants	Transcriptomes, plants	https://sites.google.com/a/ualberta.ca/onekp/
10KP 10’000 Plants	Genomes, plants	https://db.cngb.org/10kp/
ACE Antarctic Circumnavigation Expedition	(meta)genomes, (meta)transcriptomes, marine microbes	https://spi-ace-expedition.ch/
Bat1K 1’000 Bat Genomes	Genomes, all bats	https://bat1k.ucd.ie/about/
Bird10K 10’000 Bird Genomes	Genomes, all birds	https://b10k.genomics.cn/
DToL Darwin Tree of Life	Genomes, Britain and Ireland eukaryotes	https://www.darwintreeoflife.org/
EBP Earth BioGenome Project	Genomes, all eukaryotes, umbrella for many initiatives worldwide	https://www.earthbiogenome.org/
ERGA European Reference Genome Atlas	Genomes, all eukaryotes in Europe	https://www.erga-biodiversity.eu/
G10K 10’000 Genomes	Genomes, umbrella for Bat1K, Bird10K, VGP, etc.	https://genome10k.soe.ucsc.edu/about/
GAGA Global Ant Genomics Alliance	Genomes, ants	http://antgenomics.dk/
Genomic Encyclopedia of Bacteria and Archaea	Genomes, bacteria and archaea	https://phylogenomics.me/major-current-projects/geba/
GIGA Global Invertebrate Genomics Alliance	Genomes, transcriptomes, non-insect non-nematode invertebrates	http://giga-cos.org/
GlobalFungi	Fungi, ITS sequences	https://globalfungi.com/
Global Virome Project	(meta)genomes, viruses	http://www.globalviromeproject.org/
i5k 5’000 Arthropod Genomes Initiative	Genomes, arthropods	http://i5k.github.io/
iBOL International Barcode of Life & BIOSCAN	DNA barcodes plants, animals, fungi	https://ibol.org/
Kew Tree of Life Project	Flowering plants, target sequence capture	https://treeoflife.kew.org/
MOSAiC Arctic Ocean Expedition	(meta)genomes, (meta)transcriptomes, marine microbes	https://mosaic-expedition.org/
Tara Oceans	(meta)genomes, (meta)transcriptomes, plankton	https://oceans.taraexpeditions.org/
The Earth Microbiome Project	Microbial communities	https://earthmicrobiome.org/
UNITE	Fungi, ITS sequences	https://unite.ut.ee/
VGP Vertebrate Genomes Project	Genomes, 70’000 vertebrates	https://vertebrategenomesproject.org/

Microbe-focused sequencing initiatives benefit from much smaller genomes, but this is countered by orders of magnitude greater species diversity, most of which remains uncatalogued. Pioneering efforts such as the Genomic Encyclopedia of Bacteria and Archaea (GEBA) aim to systematically fill gaps in the phylogeny and to sequence type strains ( Whitman et al. 2015; Mukherjee et al. 2017). Others apply metagenomics approaches and are driven more by ecosystem ecology than phylogeny, including the Earth Microbiome Project (EMP) ( Gilbert et al. 2014), Tara Oceans ( Sunagawa et al. 2020) and other marine surveying projects. Many are driven by the impacts of microbes on human health, e.g. the Global Microbial Identifier (GMI) consortium ( Aarestrup et al. 2012) collates genomic information of microorganisms linked to epidemiological data for bacteria, viruses, parasites, and fungi, and the Human Microbiome Project that focuses on host-microbiome interactions ( iHMP Research Network Consortium 2019). Similarly, the Global Virome Project aims to improve understanding of the diversity and ecology of viral threats ( Carroll et al. 2018).

In addition to reference genomes, collections of sequence data are growing through DNA barcoding initiatives that define standardised molecular marker(s) for species identification, e.g. cytochrome c oxidase I (COX1) for metazoans, internal transcribed spacer (ITS) for fungi, 16S rRNA for bacteria, and ribulose-1,5-bisphosphate carboxylase/oxygenase (rbcL), maturase K (matK), and ITS for plants. The main reference libraries include the Barcode of Life Data (BOLD) System ( Ratnasingham & Hebert 2007) and the International Barcode of Life (iBOL) ( Adamowicz et al. 2017). Ongoing barcoding efforts, such as the iBOL consortium’s BIOSCAN programme ( Hobern 2020), continue to expand molecular sequence data collection to speed up species discovery as well as exploring species interactions and tracking their dynamics. Together, these sequence data generation initiatives aim to produce molecular catalogues with associated metadata of the entirety of Earth’s biodiversity.

*Metadata standards requirements for use in biodiversity research*

Many sequencing initiatives have and will continue to produce molecular data in the form of reference-quality genomes, complete transcriptomes, and lineage-tailored DNA barcode libraries. In terms of tangible outcomes for biodiversity knowledge, these data represent a rapidly growing comprehensive molecular ‘lookup table’ for species identification. To ensure accuracy, species must be correctly identified and recorded during sample collection and referenced to a taxonomic backbone (e.g. NCBI or GBIF), with subsequent management of reference or voucher information, and publishing with the respective voucher and taxon identifiers. To this end, sample vouchering experience from museums such as the Smithsonian has been vital in driving standards development through collaborative initiatives such as the Global Genome Biodiversity Network (GGBN) ( Droege et al. 2016). These efforts helped to extend data models for classical specimens, e.g. Darwin Core ( Wieczorek et al. 2012) and Access to Biological Collections Data (ABCD) ( Holetschek et al. 2012), in order to build a new data model for molecular sequence data. One of the key roles of initiatives like the Earth BioGenome Project and others ( Table 1) is to coordinate the development of protocols and standards for sample collection and metadata capture in line with such data models, building on established reporting standards that aim to make genomic data discoverable, e.g. developed by the genomic standards consortium ( Field et al. 2014).

In the context of infraspecific diversity conserved in plant, forest, and animal genetic resources, several projects are developing common recommendations and metadata standards to improve the conservation and sustainable use of these resources, e.g. GenRes Bridge ( GenResBridge 2021), DivSeek ( DivSeek 2021), and FAANG ( FAANG 2021). Metagenomics projects also recognise the importance of developing data standards for describing essential steps, including sampling, sequencing, data analysis, archiving, and dissemination ( ten Hoopen et al. 2017). Across the board, tools that make metadata management easier, such as the COPO platform for brokering collaborative open omics data ( Shaw et al. 2020), are helping to ensure that data are increasingly Findable, Accessible, Interoperable, and Reusable (FAIR) ( Wilkinson et al. 2016). These examples highlight the challenges involved as well as the importance of developing and applying community standards to comprehensively describe the sources of molecular sequence data collections. Good metadata management is critical to enable biorepositories to collect and preserve Earth’s genetic and genomic biodiversity in molecular sequence collections, while making it both available to and usable by researchers worldwide.

*Benefits of connecting sequencing data to biodiversity research infrastructures*

Data management frameworks aim to connect data generation initiatives to biodiversity research infrastructures in order to accelerate and expand the capabilities of existing species quantification and monitoring efforts. To achieve a unified global record of species populations in space and time, two principal Essential Biodiversity Variables (EBVs), species abundance and distribution, are required ( Jetz et al. 2019). To detect critical and potentially long-lasting biodiversity change, additional EBVs need to be prioritised such as allelic diversity, survival rates, ecosystem heterogeneity, phenology, range dynamics, size at first reproduction, and body mass index ( Schmeller et al. 2018; Kissling et al. 2018). Taxonomically annotated molecular catalogues of Earth’s biodiversity provide the means to scale up data collection of species and community EBVs that can be extrapolated from sequencing georeferenced samples. DNA barcoding has proven to be a cost-effective way of providing a model for integrating genomic data resources and biodiversity catalogues. For example, connecting GBIF with the UNITE database, a fungi-focused DNA barcoding resource ( Nilsson et al. 2019), enables spatial and temporal surveying even for ‘dark taxa’ without any physical specimens or resolved taxonomic names ( Ryberg & Nilsson 2018). Another example is the DNA barcode reference library of Canadian invertebrate fauna, which is supported by voucher specimens, digital images, and DNA extracts, with sequences deposited at GenBank and BOLD, and specimen data contributed to GBIF as Darwin Core records ( deWaard et al. 2019).

Beyond barcodes, employing the MGnify resource ( Mitchell et al. 2020) to perform taxonomic assignments of microbiome sequencing data, the European Molecular Biology Laboratory European Bioinformatics Institute (EMBL-EBI) and GBIF teamed up to facilitate the generation of sequence-based occurrence records from georeferenced European Nucleotide Archive (ENA) samples as standardised Darwin Core sampling-event datasets ( Schigel et al. 2019). Facilitating these processes is important to ensure that DNA-derived data are made discoverable through biodiversity platforms and thus increase the value of sequences with associated coordinates and timestamps ( Andersson et al. 2020). These examples show that making such connections can (i) extend traditional sampling methods of observing, capturing, or extracting, to massively scaled-up sampling using metagenomics or environmental DNA (eDNA) techniques; and (ii) transform traditional expert identification approaches into super-fast molecular species identification using progressively more comprehensive reference sequence databases. To realise these benefits, the future will therefore increasingly need to combine new sequencing technologies and bioinformatics data models for molecular sequence data management with field ecology to match metagenomics or eDNA data to reference genomic species libraries.

*Mutually beneficial outcomes for sequence collections and biodiversity infrastructures*

For biodiversity research to exploit the full value of data from molecular sequence collection initiatives, it is clear that robust and reproducible approaches to data integration are required. Ongoing efforts to coordinate traditional biodiversity infrastructures exemplify how developing common standards and practices enhance interoperability and value. For example, the DiSSCo research infrastructure works towards the digital unification of all European natural science collections ( DiSSCo 2021), and the Consortium of European Taxonomic Facilities ( CETAF 2021) brings together collections from museums, botanic gardens, and others, with a research focus on taxonomy and systematic biology. Such digitalisation and standardisation greatly facilitate the task of connecting sequence collections and biodiversity research infrastructures, exemplified by recent GBIF-UNITE and GBIF-EBI collaborations ( Andersson et al. 2020).

As well as accelerating and expanding the capabilities of existing biodiversity quantification and monitoring efforts, molecular data can support biodiversity research more widely. For example, by helping to extend, refine, and update catalogues of known species, particularly for microbes and fungi but also other groups such as insects where possibly 80% of species remain to be discovered ( Stork 2018), known as ‘dark’ biodiversity. Reciprocally, traditional biodiversity data and resources can help inform detailed annotations of sequence collections, linking data to knowledge about species biology and ecosystem compositions. One way this corpus of data from an estimated 500 million scholarly publications including all known species and their taxonomy, can be made FAIR-compliant is through the BLR ( Agosti et al. 2019) and its reuse by GBIF. Thus by making the connections, decades of accumulated learning can transform into new and refined knowledge supported by molecular data, greatly advancing data-to-knowledge research.

Here we outline current technical capabilities with respect to the tools and other resources that support the molecular components of biodiversity informatics, and present four use case examples focused on (i) sequence-informed taxonomies; (ii) ocean metagenomics; (iii) agricultural food security genetics; and (iv) global fungal diversity. These illustrate current efforts and resources to link sequence collections with biodiversity infrastructures. They inform strategies for developing national biodiversity programmes, while also highlighting key gaps that need to be addressed. Together with other examples, they help to formulate recommendations for closer integrations through ELIXIR and other infrastructures that will shape the future of biodiversity research.

### ELIXIR as an infrastructure to support integration of molecular and other biodiversity-related data

ELIXIR is an intergovernmental European organisation that brings together life science resources including databases, software tools, training materials, cloud storage and supercomputers, to connect and unite infrastructures vital for scientific research ( ELIXIR 2021a). It coordinates, integrates, and sustains bioinformatics resources across its member states, enabling users in academia and industry to access services that support scientists to exchange expertise and develop best practices, as well as to find and share the accumulating volumes of data being generated by publicly funded research. ELIXIR services (i.e. resources for users), platforms (i.e. technical domains for implementation), and communities (i.e. use cases) aim to develop and provide solutions to manage life sciences data of increasing quantity and complexity, with robust bioinformatics infrastructures and the best tools and training to drive innovation. These principles also apply to the growing field of biodiversity informatics, and it is thus timely to begin to identify the key life sciences resources, from both within the established ELIXIR infrastructures and beyond, which are required to effectively support biodiversity research. This includes the acquisition, analysis, and archival of molecular sequence data, and their integration with other biodiversity-related data and resources.

As this is a rapidly moving field, rather than listing these resources in a static table herein, we provide a contextualised list on the ELIXIR services website: https://elixir-europe.org/services/biodiversity. Over time, this portfolio of biodiversity informatics resources and services will be reviewed and extended to reflect the *status quo*, bringing visibility to existing infrastructures as well as stimulating initiatives to address key gaps and improve integration. Many demonstrate how ELIXIR already acts as an infrastructure to support the integration of molecular and other biodiversity-related data, as elaborated in the four different use cases detailed below. The current range of identified resources includes those that enable deposition and archival of molecular data as well as facilitating access to and retrieval of biodiversity-relevant data. This extends to software, workflows, and computing resources for data analysis, for improving data interoperability, and for using molecular data to address key questions in biodiversity. It incorporates access to training for researchers coming from diverse backgrounds, and advocates FAIR data principles of findability, accessibility, interoperability, and reusability as a cornerstone of any infrastructure that supports the integration of molecular and other biodiversity-related data.

In addition, wider assistance and guidance to help with Life Science data management, can also be found in the ELIXIR Research Data Management Kit ( RDMkit 2021), an online guide containing good data management practices applicable to research projects from the beginning to the end.

## Use cases: Integrating sequence collections and biodiversity infrastructures

Here we describe four use cases that demonstrate how biodiversity-relevant bioinformatics resources are being used to connect and integrate sequencing data with biodiversity-related research infrastructures to enhance interoperability and value. The use cases cover a broad spectrum with a common theme of showing examples of how these tools and other resources are used in order to process, analyse, and archive molecular sequencing data, within the broader context of biodiversity-related data generation and research. Use case 1 examines taxonomies, the key roles they play in biodiversity research, and the interdependence of molecular data and taxonomic references. Use case 2 turns to metagenomics data and the exploration of the hidden diversity of the world’s oceans. Use case 3 highlights genetics and genomics resources and initiatives for food security and agriculture. Finally, use case 4 details efforts to describe and understand the patterns of global distributions and diversity of fungi using molecular data. Although by no means exhaustive, these use cases provide clear examples of key life science tools and resources supporting biodiversity informatics through the integration of molecular and other biodiversity-related data to facilitate global efforts to protect and restore biodiversity.

### Use case 1: The interdependence of molecular and biodiversity resources via taxonomic names

**Figure 1.**
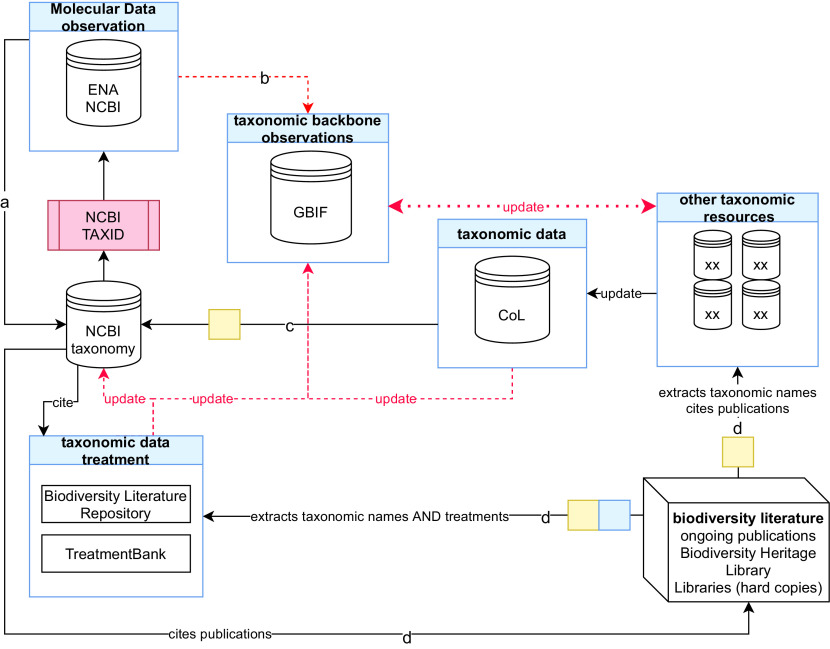
Interconnections of taxonomy and molecular data resources. A schematic representation of the primary molecular and taxonomy data resources illustrating how they are interconnected to support the development of comprehensive taxonomies linked with unique taxonomic identifiers (taxID). Specifically, each NCBI taxID is associated with a molecular sequence in (a) the NCBI and ENA primary databases which feed (b) the GBIF taxonomy backbone. (c) CoL informs both the NCBI taxonomy and GBIF with new or updated taxa names taking information from third party specialised resources. Finally, (d) literature data are used to extract taxonomic names and treatments to enhance and update NCBI taxonomy, GBIF and CoL through the Biodiversity Literature Repository and TreatmentBank. ‘XX’ indicates taxon or other specialised resources. Lines with arrows indicate data sharing efforts. Dashed lines with red arrows indicate that only a part of data is shared with the destination resources. Blue boxes highlight machine annotation and yellow boxes indicate human curation. NCBI, United States National Center for Biotechnology Information; ENA, European Nucleotide Archive; GBIF, Global Biodiversity Information Facility; CoL, Catalogue of Life.

Creating a comprehensive taxonomy linked with unique taxonomic identifiers (taxIDs) concerns mainly an efficient interoperability function in molecular biodiversity studies such as DNA barcoding and metabarcoding, phylogeny inference, genomics, and data retrieval. Occurrence and taxonomic data such as those present in the GBIF taxonomic backbone ( Figure 1a) provide the opportunity to summarise the geographical distribution of included taxa and more recently the described taxa supplied by BLR ( Agosti et al. 2019). However, such data are not necessarily linked to unique taxIDs across repositories and might include several synonyms that also remain unlinked. The same issue can be encountered in the CoL ( Roskov et al. 2020) resource where the most recent recognised taxonomy, when covered, is reported ( Figure 1b). It is important to note that not all these taxonomic entries have associated molecular sequence data where many of them originate from classical biodiversity studies. In this context, while seeking a new experimental design for molecular characterisation of specific organisms, the absence of unique identifiers (i.e. taxIDs) represents an important issue in collecting the most comprehensive information related to the organisms of interest. This may be due to several reasons including the presence of synonymous names, taxa with the same scientific names but with different taxonomic classifications, the splitting of well-established species leading to the nomination of new and different taxa, e.g. European Grass Snake ( Kindler et al. 2017), or evidence-based renaming of species, such as the fungus that causes ash dieback ( Baral et al. 2014), requiring additional needed legacy information to track the recent changes in taxonomic classifications and link them efficiently to a reference taxonomy.

The NCBI taxonomy database ( Federhen 2012; Schoch et al. 2020) offers well-structured taxonomic classification reports in which ‘synonyms’ and ‘equivalent names’ are linked to the unique taxID of the main taxon scientific name ( Figure 1a). In particular, the release of February 10th 2021 contains 179,314 declared synonyms and 1,180 scientific names with more than one taxonomic path or rank. For instance, *Diplura* is the scientific name of both order and genus ranks, *Centipeda* is a genus name belonging to plants and firmicutes, and *Taenidia* is a Coleoptera subgenus and a genus name belonging to plants. As noted above, the lack of molecular sequence data for many established taxa means that they currently have no corresponding NCBI taxIDs. This represents the gap between the NCBI taxonomy and other repositories or backbones such as CoL and GBIF ( Figure 1b and c). In addition, other molecular sequence collections, such as BOLD, contain entries with related taxonomic information sometimes not yet incorporated into the NCBI taxonomy and consequently lacking unique NCBI taxIDs.

An important way to enhance the completeness of the taxonomy information is to merge and harmonise such information coming from different sources. A good example in this context is the EukMap platform developed within the UniEuk project ( Berney et al. 2017). It is an open-source software currently oriented to protist taxonomy management, but it can be deployed by other communities adapting it to their needs. The platform adopts an online open collaboration concept for expert driven curation able to link state-of-the-art phylogeny-based taxonomy with genetic information. As such, taxonomists are encouraged to propose updates or corrections to the taxonomy using the platform. Proposals are then validated by community experts to feed into the official release of the UniEuk taxonomic framework with the goal of pushing these changes to the common taxonomy resources such as the NCBI ( Schoch et al. 2020) and SILVA ( Quast et al. 2012).

More generally, a solution for taxonomic name integration is included in the published literature ( Figure 1d). All these names including their history are documented in the huge, daily growing corpus of highly structured taxonomic literature, comprising well over 100M pages of printed or more recently electronically published literature, dating back to the origin of modern taxonomy in 1753 and 1758 for plants and animals respectively ( Linné & Salvius 1753, 1758). Each taxonomic name is accompanied by a taxonomic treatment with a description and/or diagnosis, notes on behaviour, distribution, vernacular names, and citations of previous treatments or synonyms. The latter functions not only similar to a bibliographic citation for articles, for which a Digital Object Identifier (DOI) can be mined, but can also be typed, for example by creating a synonym (see e.g. the original description of the honey bee *Apis mellifera* by Linnaeus ( Linnaeus 1758). In this last issue, text mining techniques would play an important role in collecting the relevant information from scientific literature to update the knowledge needed to resolve such ambiguity. For example, Plazi ( Plazi 2021) extracted over 370,000 taxonomic treatments and data therein including taxonomic treatment citations ( Miller et al. 2015). These data are FAIR and reused by GBIF and accessible through Plazi’s application Synospecies, providing access to the taxonomic names and synonyms as linked open data. They are also submitted once a day to NCBI, albeit only data covering organisms already present in the database, and thus morphological based species without molecular sequence depositions are discarded.

An additional source of information on taxon names used in scientific publications falls outside taxonomic treatments, such as linked supplementary data tables (e.g. listing all sequenced specimens with their corresponding taxonomic names and accession numbers), or a list of species or molecular taxonomic units identified from a metabarcoding survey ( Figure 1d). Clearly the advantages of having access to the taxonomic treatments and to the structured data tables embedded in the scientific papers, as this allows understanding the reasoning for creating a new species name or synonym, are numerous. This also provides access to cited specimens, permits the discovery of advanced species/taxa interactions such as viral hosts or plant pollinators, and promotes the development of a harmonised and complete list of taxonomic names tagged by unique taxIDs.

### Use case 2: Metagenomics exploration of the hidden diversity of the world’s oceans

Biodiversity data derived from marine metagenomics datasets have grown substantially during the last years and can serve as an excellent example of how molecular sequence data have expanded the insight and understanding of microbial biodiversity in the marine environment. Before the establishment of ELIXIR ( Harrow et al. 2021) and the Marine Metagenomics Community ( MMC 2021), there was a lack of standards on how to process the data and deposit metagenomic and metagenomic-derived data into appropriate databases. As one of the first steps to address these gaps, the MMC published best practices ( ten Hoopen et al. 2017) that served as a foundation for a community standard to enable reproducibility and better sharing of metagenomic data along with comprehensive sampling metadata. As a part of the work, the community built and benchmarked analysis pipelines, established domain-specific reference databases and established better procedures for deposition of metagenomic data.

**Figure 2.**
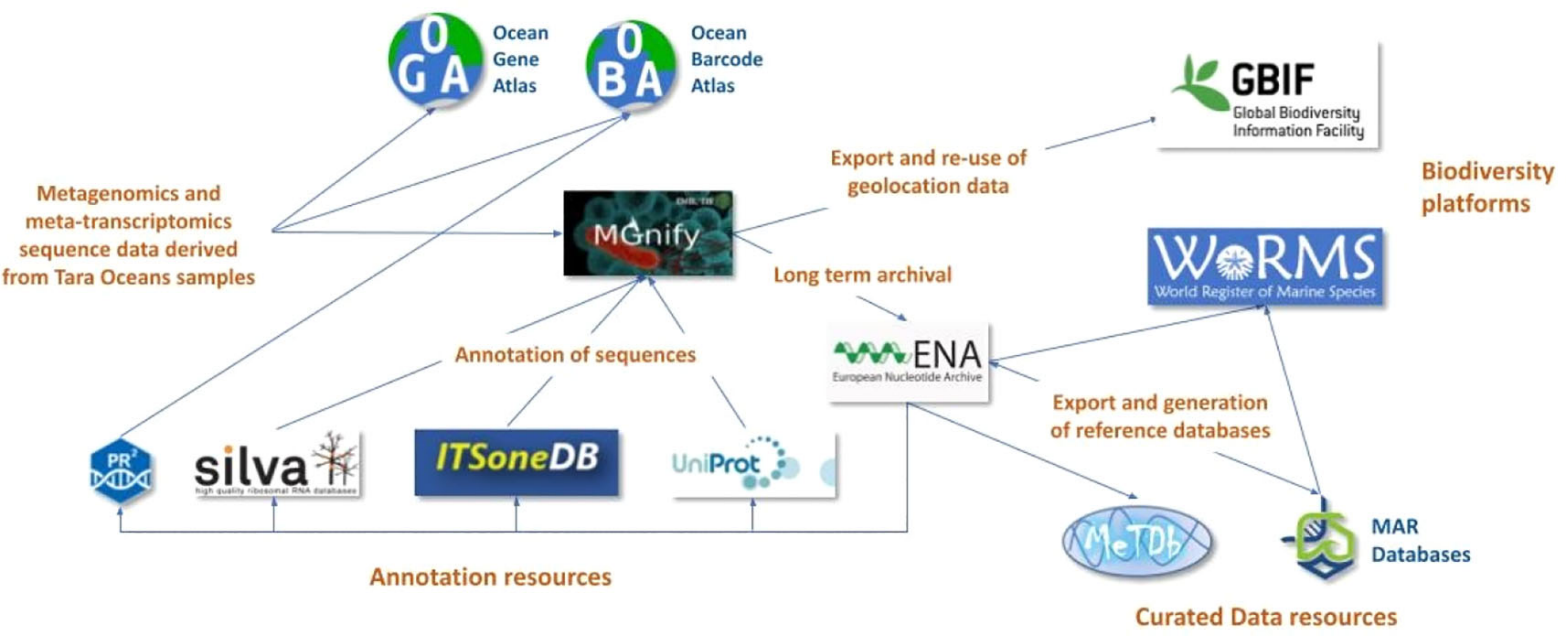
Overview of the processing of marine environmental sequence information. A simplified flowchart of the processing steps of information from the Tara Oceans datasets (metagenomics, metatranscriptomics, and amplicons) to integrate data with primary and secondary resources and other biodiversity platforms as GBIF and WoRMS. Processing of sequence data from oceanic water samples using informatics tools and services connects them with taxonomic information and links them to knowledge about species biology and ecosystem variables. PR ^2^, pr2-primers: an 18S rRNA primer database for protists; SILVA, an on-line resource for quality checked and aligned ribosomal RNA sequence data; ITSoneDB, a collection of eukaryotic ribosomal RNA Internal Transcribed Spacer 1 (ITS1) sequences; UniProt, the universal protein resource of sequence and functional information; ENA, European Nucleotide Archive; MGnify, the microbiome analysis resource; OGA, Ocean Gene Atlas; OBA, Ocean Barcode Atlas; GBIF, Global Biodiversity Information Facility; WoRMS, the World Register of Marine Species; MetDB, a genomic reference database for marine species; MAR databases, a collection of richly annotated and manually curated contextual and sequence resources for marine species.

An example project that has been very successful in using molecular sequence data to inform and enrich our understanding of biodiversity is the work undertaken by the Tara Ocean Foundation ( TARA 2021). Within this project, several major studies have been undertaken since 2009 using molecular sequencing techniques to characterise the life of the world’s oceans. Tara Oceans has advanced our knowledge of all microbial kingdoms of life present in the ocean, from bacteria to small eukaryotes, as well as viruses (e.g. 150,000 eukaryotic taxa in the epipelagic ocean at 90% unknown, nearly 200,000 new double-stranded DNA viruses). The approach uses meta-barcoding, metagenomic and meta-transcriptomic data sequencing ( Sunagawa et al. 2020) to generate large numbers of raw sequence reads derived from organisms present in water samples. The Ocean Gene Atlas ( Villar et al. 2018) is a web service to explore the biogeography of marine genes ( Figure 2) based on sequence similarities and consists of the Tara Ocean Microbiome - Reference Gene Catalog database (OM-RGC) and the Marine Atlas of Tara Ocean Unigenes (MATOU) ( Sunagawa et al. 2015; Tara Oceans Coordinators et al. 2018). The OM-RGC contains 46 million bacterial/archaeal genes, generated from metagenome raw data, while MATOU contains 117 million eukaryotic genes, generated from the metatranscriptome raw data. The raw data from Tara Oceans has also been submitted to MGnify - a free to use resource for analysis, visualisation and discovery of metagenomic, metatranscriptomic, amplicon and assembly datasets ( Mitchell et al. 2020). Approximately 1,300 samples in eight studies have so far been analysed in MGnify, including 370 metatranscriptome and metagenome samples. Of these, 1,189 amplicon events have been registered in GBIF, giving rise to more than 750,000 biogeography occurrences ( GBIF 2018). The sequence datasets analysed in MGnify are stored in the European Nucleotide Archive (ENA) and re-used in other marine reference databases such as METdb, a genomic reference database dedicated to micro-eukaryotic species, containing 348 organisms and 463 strains of micro-eukaryotic species derived from transcriptome sequence data ( Niang et al. 2020).

The metagenome-assembled genomes (MAGs) generated from analyses of shotgun sequenced samples in MGnify have been included in the MAR databases ( Klemetsen et al. 2018), a collection of richly annotated and manually curated contextual (metadata) and sequence databases for marine prokaryote species. Context is captured through ensuring compliance with the Genomic Standards Consortium ( Field et al. 2014) recommendations for Minimum Information about any (x) Sequence (MIxS) standards, an overarching framework of sequence metadata ( Yilmaz et al. 2011). These resources are accessible through the Marine Metagenomics Portal ( MMP 2021), with the MarRef containing nearly 1,000 complete microbial genomes, and MarDB hosting more than 13,000 non-complete genomes. The MAR database entries are cross-referenced with ENA and the World Register of Marine Species (WoRMS) ( Vandepitte et al. 2018) records to ease the access to additional and curated metadata. The data from the Tara Oceans project also provides links to several other databases such as UniProt ( The UniProt Consortium 2019), a high-quality curated database of protein sequences and functional information, SILVA ( Quast et al. 2012), a database for ribosomal RNA (rRNA) genes used for phylogenetic reconstruction, PR ^2^ ( Vaulot et al. 2021) a reference database of 18S rRNA protist sequences carefully curated by experts from each taxonomic group in the context of EukRef project, and ITSoneDB ( Santamaria et al. 2018), a specialised collection of ribosomal RNA Internal Transcribed Spacer 1 (ITS1) sequences aimed at the taxonomic identification of eukaryotes. The Ocean Barcode Atlas (OBA) is a web service designed to explore the biodiversity and biogeography of marine organisms at planetary scale for Tara Oceans and other marine metabarcode datasets ( Vernette et al. 2021).

Figure 2 illustrates how raw environmental sequence data derived from oceanic water samples are processed, annotated, and re-used, applying informatics tools and services to connect them with taxonomic information that helps link the data to knowledge about species biology and ecosystem variables. These sequence datasets therefore serve as a measure to determine diversity and abundance in a specific habitat, provide a means to quantify declines in biodiversity and climate change, and allow for efficient comparisons of datasets, e.g. time-series experiments, in environmental or species monitoring programmes.

Other large-scale projects to analyse ocean biodiversity have also been undertaken in recent years, including the Malaspina expedition ( Duarte 2015), Ocean Sampling Day initiatives ( Kopf et al. 2015), the Antarctic Circumnavigation Expedition ( ACE 2021), and the Multidisciplinary drifting Observatory for the Study of Arctic Climate expedition ( MOSAiC 2021). On the one hand, the large and growing variety of observations taken during oceanic sampling ( Gorsky et al. 2019) have posed many data management challenges. On the other hand, facing these challenges means that the field of marine metagenomics has paved the way towards better capturing, processing, and managing of samples and their metadata. In parallel to these studies addressing diversity issues at the global ocean scale, smaller spatial scale studies addressing temporality issues have emerged (including classical diversity data, genomic data, and imaging data) on enhanced marine genomic observatories ( Bourlat et al. 2013; Davies et al. 2014). More generally, increasingly integrative approaches to diversity analysis are now favoured by the marine research community ( Canonico et al. 2019; Collins et al. 2020). Although marine metagenomics is relatively mature as a field, there are still many issues that need attention. There is a need to implement standardised procedures for processing and analysing datasets, including best practices for assembly, binning and annotation. Furthermore, the quality of reference databases, integration of new omics data, specific data warehouses, and long-term data management services are issues that warrant careful attention, e.g. in the context of moving from biodiversity snapshots to large-scale monitoring and discovery.

### Use case 3: Biodiversity genetics and genomics for food and agriculture

**Figure 3.**
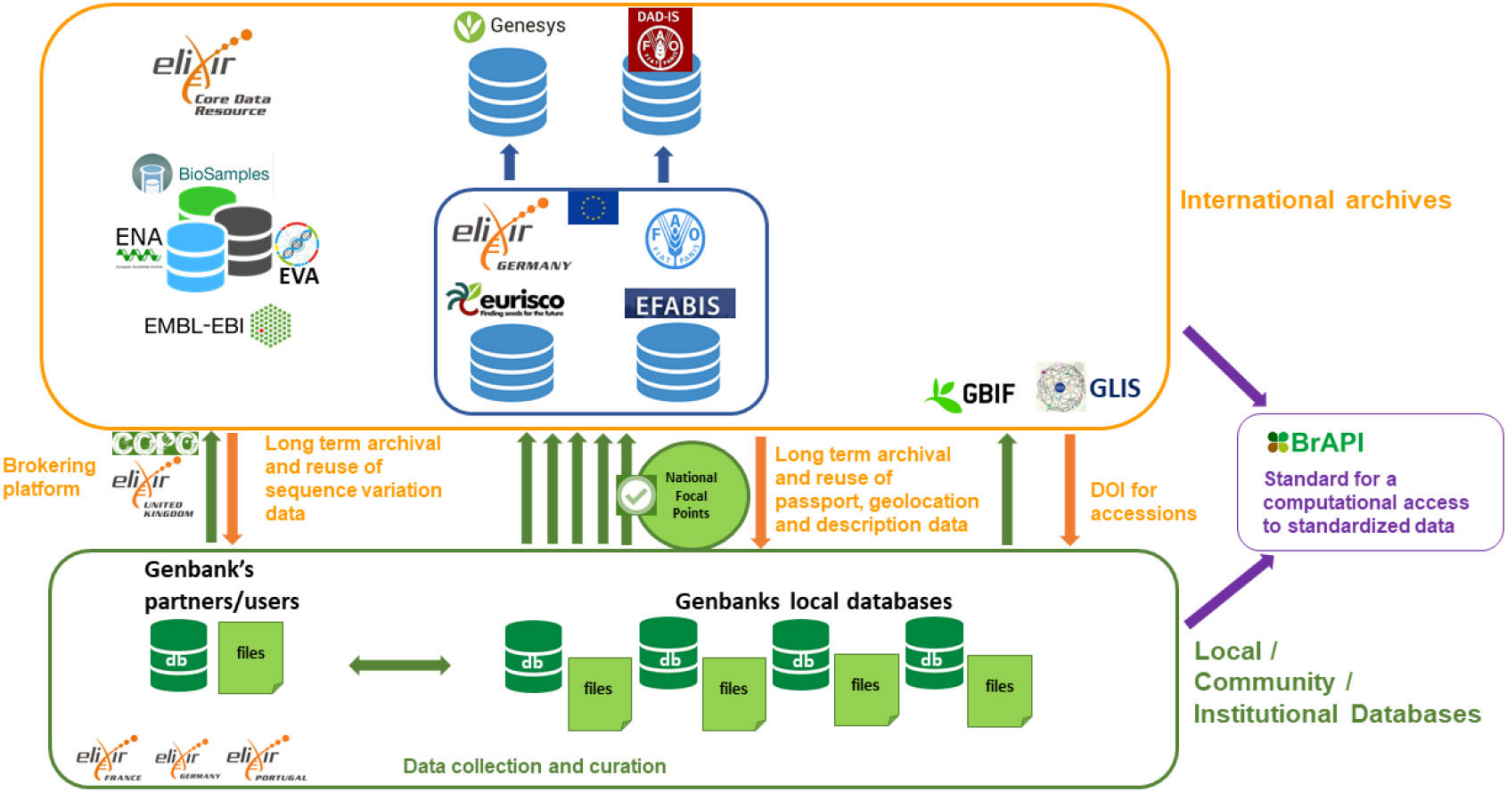
Overview of the main information systems used for archiving data on genetic resources for food and agriculture. In the green box at the bottom, the information systems used to manage the data collected and curate it. Some of these information systems are maintained by ELIXIR nodes in their national infrastructures. The data can then regularly be submitted and updated in international archives. The list of Genetic Resource accessions are archived in the European Search Catalogue for Plant Genetic Resources (EURISCO) and the European Farm Animal Biodiversity Information System (EFABIS) after clearance by National Focal Points appointed by country governments and then collected by global information systems, Genesys and the Domestic Animal Diversity Information System (DAD-IS). They can also archive their datasets at GBIF without any clearance. Genotyping and genomic data are archived in ELIXIR deposition databases and Core Data Resources (EMBL-EBI ENA, EVA and BioSamples). Brokering platforms such as COPO, can be used to facilitate data submission to international archives. ELIXIR has also contributed to a global standard for a RESTful application programming interface (API) focused on plant data, BrAPI, that is progressively implemented on the main plant information systems to allow automatic access to standardised data. GBIF, Global Biodiversity Information Facility; ENA, European Nucleotide Archive; EVA, European Variation Archive.

Adaptation of agriculture has been based on fitting crop varieties and breeds to their production system, which includes farming systems and their natural environments. This has led after initial domestication to the development of a large diversity of varieties and breeds adapted to local farming conditions but also to diverse usage and consumer demands. With the specialisation and industrialisation of production systems after the Second World War, this high intraspecific diversity has started to decline all over the world and is now threatened in many cases ( Pilling, Bélanger & Hoffmann 2020b). Important initiatives to catalogue and conserve this diversity in large *ex situ* collections or with participatory *in situ* approaches have grown in parallel with a global governance under the auspices of the United Nations Food and Agriculture Organisation (FAO) ( Pilling, Bélanger, Diulgheroff, et al. 2020a). The global objectives of these initiatives are to secure this biodiversity as the indispensable foundation of sustainable food production systems ( Smale & Jamora 2020), highlighted in the EU Biodiversity 2030 Strategy, the EU Green Deal, and the UN Sustainable Development Goal 2.5 (Zero hunger - maintain the genetic diversity of seeds, cultivated plants and farmed and domesticated animals and their related wild species). The global collections of genetic resources, comprising ~5.4 million plant accessions from over 50,000 species and over 7,800 local breeds ( Pilling, Bélanger, Diulgheroff, et al. 2020a), are managed by a large number of stakeholders. Plant genetic resources are conserved in more than 700 genebanks from 103 countries and 17 regional or international research centres ( Pilling, Bélanger, Diulgheroff, et al. 2020a), that contribute to international catalogues of genetic resources such as the European Search Catalogue for Plant Genetic Resources (EURISCO) ( Weise et al. 2017) and the European Farm Animal Biodiversity Information System (EFABIS) at the European level and the Domestic Animal Diversity Information System ( DAD-IS 2021) and GENESYS ( Genesys 2021) at the international level ( Figure 3; ( FAO 2010)). Another possibility for archiving data on collections of genetic resources is to use the GBIF portal, which is often carried out in parallel as an alternative that does not require any clearance by governmental agencies ( Figure 3; e.g. datasets at GBIF from The Netherlands Centre for Genetic Resources, ( Menting 2020)).

Since the 1980s, collections of accessions have been genotyped with a set of fast-evolving techniques, mainly to understand crops and breed evolution since domestication and for the identification of adaptive traits, e.g. ( Wilkinson et al. 2013; Brozynska et al. 2016; Mascher et al. 2019). Sequence variation has also proved useful and is increasingly used for monitoring current maintained genetic diversity, e.g. detection of redundancy in collections, or assessment of threat levels facing small populations of breeds, forest trees or crop wild relatives ( Bélanger et al. 2019). International archives have been developed to store sequence variation data, such as dbSNP ( Sherry et al. 2001) focused on Sequence Nucleotide Polymorphisms (SNPs) or the European Variation Archive ( EVA 2021) launched more recently to store any type of sequence variant that can be expressed in Variant Call Format (VCF) ( Figure 3). Companion archives can be used to track the accession identifiers and collection provenance (BioSamples and BioStudies at EMBL-EBI or BioProjects at NCBI) while the reference genomes used for the detection of the variations must be stored in the INSDC archives prior to the submission of variation data ( Figure 3).

A key challenge to be addressed in the context of biodiversity genetics and genomics for food and agriculture rests with the identification of the accessions of genetic resources and their consistency across the catalogues and molecular archives. Breeds or crop variety names that are critical information for linking data obtained on genetic resources to previous knowledge are also a challenge for interoperability due to misspelling, homonyms and synonyms over time, and across regions and borders. Given that reference genome sequences, sequence variation data and catalogues of accessions of genetic resources are usually managed by separate groups, they often end up in different silos with poor or no interoperability. This also affects the interoperability of the data once submitted to international archives, which is still not a routine practice. It is therefore currently not possible to automatically obtain the genetic variation data associated with a given panel of accessions selected in a catalogue of genetic resources or reciprocally, to retrieve all known information on the origin of the accessions (country of origin, type of material, etc.) associated with a variant found in EVA or dbSNP. For crops, the United Nations FAO recently recommended adding a DOI to the three fields of the MultiCrop Passport Data standards that have ensured the unique identification of accessions to date (species name, holding institution name, and the accession identifier provided by the holding institute) and developed a dedicated service (GLIS: the Global Information System on Plant Genetic Resources for Food and Agriculture, Figure 3) to support the adoption of this new practice by genebanks. The communities working on crops, farm animals and forest trees are also actively working with EMBL-EBI to develop dedicated specifications for the metadata associated with the data archived in ENA and EVA and in particular to ensure that they track identifiers associated with accessions of genetic resources. In this context it is important to take into account the possible different scales at which the genetic resources are collected, i.e. an individual for most crops, and populations for breeds and most forest trees. Reciprocally, mechanisms for capturing and updating in genebank catalogues the identifiers associated with the samples that were genotyped or sequenced are needed (see e.g. in relation with domestic animals biological resources: IMAGE 2021). These challenges are not necessarily unique to biodiversity genetics and genomics for food and agriculture, but they particularly highlight efforts required to informatically process and connect sequence data with sample metadata.

### Use case 4: Understanding biogeographical diversity through molecular mapping of global fungal distributions

**Figure 4.**
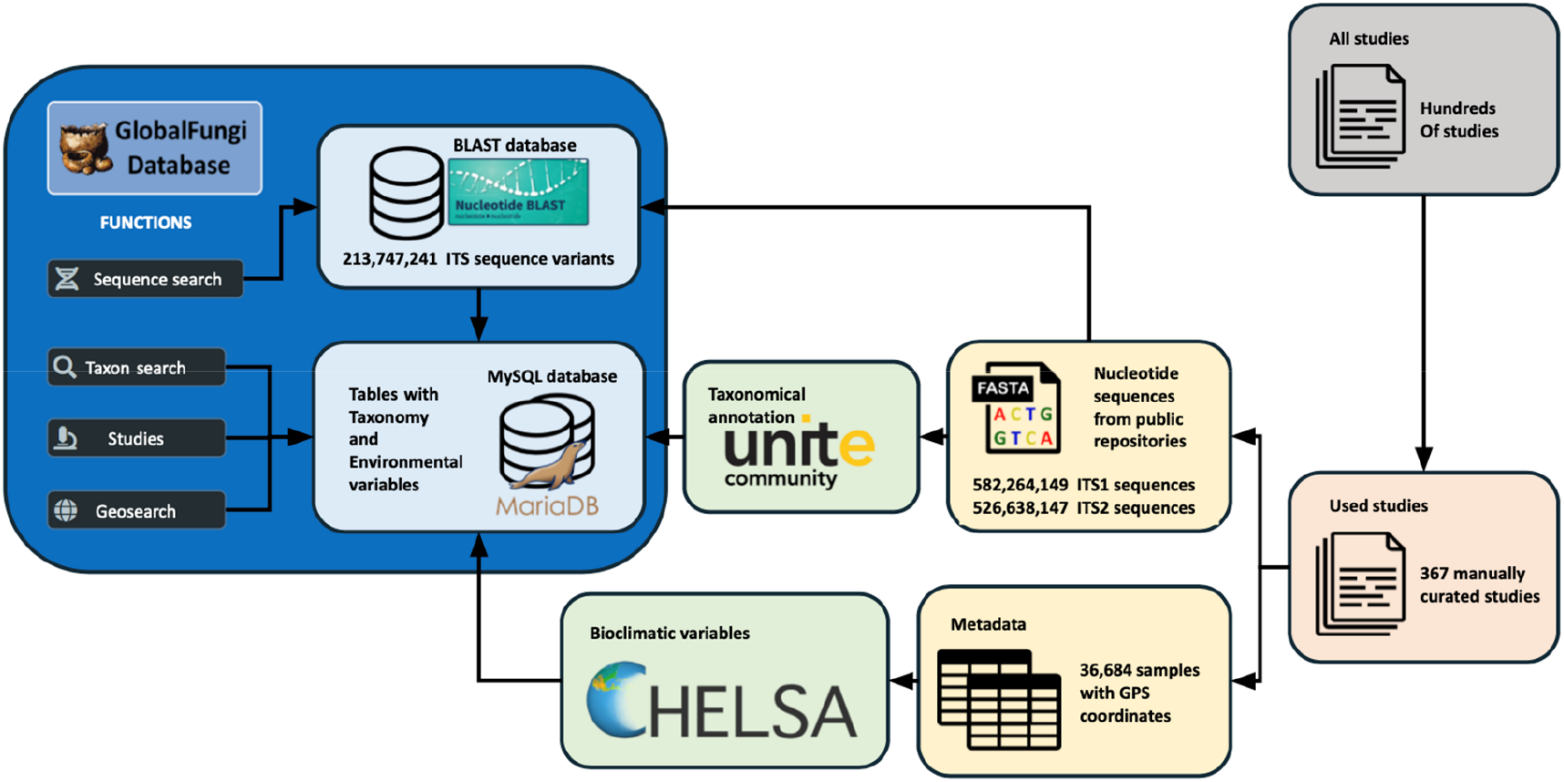
Data and annotation sources connected via the GlobalFungi Database. GlobalFungi enables searches for specific sequence variants, fungal genera, species, and molecular species (called ‘species hypotheses’) by performing BLASTn sequence searches and querying a local MySQL database. Annotation of taxa is based on UNITE, the database of fungal molecular taxa. Samples can be queried by searching on maps or through the studies where they were published. Climatic data for sample locations are retrieved from the CHELSA (Climatologies at High resolution for the Earth’s Land Surface Areas) database. ITS, Internal Transcribed Spacer; GPS, Global Positioning System.

The GlobalFungi Database ( GlobalFungi 2021) exemplifies efforts to connect sequencing data to biodiversity research infrastructures and advance data-driven research. Fungi play key roles in all terrestrial ecosystems, primarily as decomposers of organic matter but also as pathogens or symbionts. Long-standing scientific interests in describing and understanding the patterns of global distributions and diversity of fungi mean that sequencing initiatives have led to an accumulating wealth of fungal molecular data from various geographical regions, ecosystems, and habitats. Large-scale studies focusing on soil fungi have used metabarcoding analysis to examine the ecological drivers and biogeographic patterns of fungal community composition and diversity ( Tedersoo et al. 2014; Egidi et al. 2019). However, coordinated global sampling at sufficient spatial and taxonomic resolution remains largely unfeasible for individual research studies. Instead, a meta-approach is needed to collect, collate, categorise, and centralise existing data using infrastructures that can continue to gather and include new and future genetic and genomic datasets. The GlobalFungi Database was established as a platform to address these needs by providing public access to published data on fungal community composition obtained by next-generation-sequencing approaches through a web-based interface that promotes FAIR principles and allows various queries and visualisations of the results ( Větrovský et al. 2020). Release version 3.0 contains over 1100 million observations of fungi from 367 manually curated studies with over 36,000 samples and 213 million ITS sequence variants ( Figure 4). GlobalFungi allows searching for specific sequence variants, fungal genera, species, and molecular species (called ‘species hypotheses’) by performing BLASTn sequence searches and querying the local MySQL database. Annotation of taxa is based on UNITE, the database of fungal molecular taxa compiled using direct sequencing of known fungal species and environmental sequencing of targeted barcodes ( Nilsson et al. 2019). GlobalFungi contains data from high-throughput sequencing efforts including local abundance of fungi and complete sampling metadata, and allows querying of samples by location searches on maps or through the studies where they were published. These are complemented with extensive climatic data for sample locations retrieved from the CHELSA (Climatologies at High resolution for the Earth’s Land Surface Areas) database ( Karger et al. 2017). The GlobalFungi Database aims to continue to grow by adding more records and by motivating the community to submit new datasets to help build the resource for research on the systematics, biogeography, and ecology of fungi.

The utility of such a centralised resource connecting sequencing data to biodiversity research infrastructures is demonstrated generally through the characterisation of global patterns of fungal biodiversity ( Větrovský et al. 2019) or predicting the global biodiversity of fungi ( Baldrian et al. 2021) and specifically through the ability to identify fungi that are carried across continents along with introduced plants ( Vlk et al. 2020). Moreover, the metadata-rich resource helped to show that symbiotic fungi are more vulnerable to climate change than pathogens and that climate change thus represents a considerable threat for forestry production, agriculture, and food security ( Větrovský et al. 2019). Beyond community diversity and biogeography patterns, the distributions of individual fungal species are particularly important, e.g. for phytopathogenic fungi that may severely affect yields of agricultural crops such as the *Fusarium* pathogen of bananas ( Dale et al. 2017). Exploiting the GlobalFungi Database, mycologists, ecologists, or global climate change scientists are able to link fungal occurrence and diversity data with the panel of collected metadata, allowing for the characterisation of key environmental factors that are driving fungal diversity. Such studies can be performed at different geographic levels, from country scales to biomes of the entire world, and for all identifiable fungal communities or for selected ecosystem compartments. Collating these data involves manual curation of information from published studies (367 studies in the latest release), but metadata heterogeneity means that attributes extracted from the publications that are common across the database are limited to just Longitude, Latitude, Continent, Sample type, Biome, Sampling year, Primers used, and pH, while additional metadata only exist for some of the studies. Nevertheless, these resources bring together different data types to enable assessments of fungal diversity across the globe and tracking of individual species or genera, leading to the development of a more comprehensive understanding of the biogeography of fungal diversity. Importantly, this also facilitates assessments of potential threats faced by fungal communities and the ecosystems of which they form such a vital part.

## Informing strategies for large-scale national biodiversity programmes

Now that large-scale regional, national, and global biodiversity genomics projects are a reality, it is vital to capitalise on the lessons learned and best practices developed through initiatives such as the example use cases presented above. The greatest impact that genetic and genomic data can have on biodiversity assessments, monitoring, conservation, and restoration will only be realised with the support of infrastructures that facilitate the finding, sharing, and connecting of increasingly large and diverse datasets. This requires efforts at all levels to be put into practice from the start, informing strategies for biodiversity programmes to ensure that the data they generate are findable and interoperable. A huge amount of data is being produced globally, and whilst the situation is improving with respect to open access for sequencing data at least, much of this data is still not made available to the research community with adherence to the FAIR principles. By developing strategies and supporting infrastructures that make this easier and scalable, usability and impact will be greatly extended: a major goal of ELIXIR. National biodiversity sequencing efforts can be a useful opportunity to demonstrate how project-wide strategies for harmonisation and standardisation of FAIR data can be put to good effect.

The primary products of these programmes, the assembled genomes and their corresponding annotations, are the fundamental building blocks that modern computational comparative approaches exploit to learn about the biology and evolution of the species ( Zoonomia Consortium 2020). These benefit from and build on accumulated knowledge from field and wet lab research compiled by biologists working on their organisms of interest and documenting experiment details and sample information. This species, experiment, and sample metadata is vital to contextualise the production of a genome and its annotation, and even more so when subsequently exploiting these resources, e.g. through gene expression analysis and interpretation using transcriptomic and other techniques.

Standardisation is essential for the successful scaling up of these initiatives. Whilst the superset of metadata used to describe biological entities and processes might be ever-expanding, metadata about the provenance of samples can be reduced to a subset of ‘core’ terms that reflect descriptions that are fundamental to the downstream contextualisation of a given sequence. For example, the Darwin Tree of Life project ( DToL 2021) is a large programme that aims to understand the biodiversity of the British Isles, by sequencing the DNA of all the animals, plants, fungi, and protists, comprising approximately 60,000 species. As a partner of the Earth BioGenome Project ( Lewin et al. 2018), DToL has worked with sample collectors who are, or collaborate with, taxonomic experts to develop a core standard for sample metadata collection alongside Standard Operating Procedures for physical preservation of samples and subsequent sequencing. The breadth of the genomes that will be produced from the wide array of habitats, collection methods, and variety of recorded traits across taxonomic groups is a key challenge in terms of ensuring compliance with these standards.

DToL is also undertaking widespread DNA barcoding of specimens. DNA barcoding contributes to rapid identification of biological material and, in terms of cost-benefit, knowing when to barcode and/or genome sequence a specimen could be seen to be a balancing act when considering how to efficiently make assessments of biodiversity. As noted in Use Case 1, barcoding provides a fast and cost-effective technical process to ascertain a provenance trail for a given organism with respect to its taxonomic lineage, an essential part of biodiversity studies. This becomes increasingly important where taxonomic identification is still uncertain due to conflicting or a lack of information, i.e. where an expert identification results in naming differences, lack of defined lineages of less well-studied organisms within the taxonomy databases, and discrepancies with taxonomic identifier allocation services such as the NCBI. As part of DToL, specific metadata schemas are being prepared to assist with the collection of standardised barcoding data alongside methodologies to automate taxonomic identification based on amplicon sequences. Data management tools incorporate and link the deposited sample metadata and the subsequent genomes in the EMBL-EBI Biosamples and ENA databases, respectively, and will also submit to BOLD.

Other national projects focus on within species diversity rather than between species. The national Swedish conifer programme to sequence the Norway spruce and Scots pine genomes serves as an example of what can be done with a well-assembled and annotated genome ( Nystedt et al. 2013). Around 75% of Sweden’s area is covered with forest, and much of this is conifer. To improve production and to inform a sustainable forestry practice, the genomes will serve as a basis for a massive resequencing effort where thousands of individuals are sequenced using short read technologies. This will in turn be used to study population structure, and tens of thousands of individuals with known phenotypes will be genotyped. These phenotypes can then be coupled to genotypes and used to improve productivity and to create varieties more adapted to climate change. This also opens up the possibility of pangenomics, an area that is growing in popularity and usefulness, especially in the context of food security highlighted by use case 3, and particularly for crop and livestock improvement ( Tao et al. 2019; Khan et al. 2020; Danilevicz et al. 2020; Della Coletta et al. 2021). To fully exploit the massive amounts of sequence data produced, they will need to be deposited with carefully annotated metadata, and stable identifiers that are coupled with phenotypic information.

Whilst the DToL and the Swedish conifer projects are at different ends of the spectrum in terms of breadth and depth, they highlight direct commonalities. They both comprise important first steps for future biodiversity studies, i.e. they develop fundamental genomic baselines on which to build future comparisons amongst organisms and populations through resequencing efforts. However, differences in sampling, naming conventions, sequencing dataset quality and coverage, and annotation quality can all lead to barriers to uptake within the FAIR data ecosystem. By using standardised methods and tools for metadata and data capture and processing, one of the key gaps in biodiversity data management is fulfilled and directly coupled to efforts to produce sequencing and barcoding data based on consistent rich metadata about the biological material from which data are derived. Technical tools, including COPO ( Shaw et al. 2020), an ELIXIR roadmapped data brokering resource, are being employed to aid consistent deposition of project-compliant data and metadata in DToL and other upcoming national and international programmes. The aim is to provide a comprehensive overview of the history of the sample, evidence for its characterisation, and its genome which is ready to be used for annotation and further study.

The DToL and the Swedish conifer projects here serve as examples, in many respects paving the way for emerging initiatives such as the European Reference Genome Atlas ( ERGA 2021). Being able to link the sampled biological material to the metadata about the collection process, the identification strategy, the sequence data, and subsequent metrics for assembly, and finally the annotation, will fill crucial gaps in FAIR data delivery in these projects. In this way, the coordination of infrastructure alongside coordination of sampling and characterisation processes based on metadata specifications is a powerful way of linking FAIR data to the methodologies that communities use to undertake biodiversity research and discovery.

## Common challenges faced when connecting molecular sequence and biodiversity research infrastructures

The four use cases and examples of large-scale national biodiversity programmes outlined above present different aspects of how infrastructures can be involved in and support biodiversity studies. They represent data and knowledge ecosystems of connected and complementary information systems. The technical solutions to overcoming data integration challenges are often somewhat domain-specific. Nevertheless, analogies can be drawn amongst the different steps taken to address specific challenges, revealing common gaps in tools and infrastructures focused on taxonomy, metadata, and community services. Cross-domain recognition of these gaps is important to ensure coordinated efforts to address priority issues that will facilitate continued commitments to open science and increased usability of biodiversity related data in support of increased research efficiency.

### Missing taxIDs, conflicting taxonomies, and information locked in publications

The informatics processes designed to connect information from biodiversity research infrastructures with molecular sequence data collections are often hindered by the inconsistent use of taxIDs across collaborating partners. For molecular sequence data, taxIDs are issued by NCBI but only for taxa where sequences have been deposited, whereas biodiversity infrastructures often employ their own distinct sets of taxIDs. Missing and non-matched taxIDs give an incomplete and inconsistent view of currently documented taxa, which greatly decreases the power of computational analyses and severely limits cross-infrastructure interoperability. Conflicting and/or not regularly updated taxonomies employed by the different infrastructures further hinder interoperability, promoting the building of data silos by distinct research communities. Furthermore, different names are currently accepted (able to be processed) by the different infrastructures, and synonym lists are not complete or not compatible. A similar situation exists for agricultural catalogues of genetic resources, where accessions, lines, and samples may be assigned conflicting identifiers by different laboratories. Moreover, the names of breeds and varieties to which they belong are not standardised, meaning that when data are shared or archived their future reuse can be limited by the uncertainty of their origins. The information necessary to address these issues exists, but is difficult to obtain as it essentially implies determining the provenance of a name. It is trapped in the collective wisdom of experts and their publications, and thus must first be extracted, e.g. using text-mining and expert curation, and then fed into reference taxonomic infrastructures with stable backbones and fully traceable identifiers. However, this does not extend well to metagenomics-focused research where ‘dark taxa’ vastly outnumber described diversity, and thus pose additional challenges in the context of defining and employing interoperable identifiers. Communities recognise that taxonomies are not static because our ever-improving understanding of life on Earth necessitates constant revisions. They also recognise that gaps created by failing to develop and support harmonisation initiatives are holding back advances in biodiversity research.

### Inconsistent metadata standards: adoption of best practices

Comprehensive and accurate recording of metadata are critical for data reuse and interoperability, but they require considerable extra efforts and cannot be rigidly enforced. They not only enable the tracing of the origins of samples or sample-derived molecular data, but they also provide the necessary context to be able to link these to other relevant data. The scope of such other relevant data could cover taxonomy, ecology and life history, climatology, biogeography, essential and extended sets of biodiversity variables, and much more, but only if the data can be correctly linked. The use cases outlined above highlight just how heterogeneous metadata can be across different research domains, but also how important it is to be able to maintain correct links in order to achieve meaningful research outputs. Metadata is particularly important in the context of connecting molecular sequence data to biodiversity research infrastructures, especially with expanding collections of molecular sequence data and efforts to build reference genomic species libraries. Although a suite of relevant metadata standards exist, e.g. Darwin Core ( Wieczorek et al. 2012) for species observations, specimens, samples, and related information, and MIxS for Minimum Information about any (x) Sequence ( Yilmaz et al. 2011), they are not used consistently and different standards are adopted by different infrastructures. This is a common problem, as research communities and projects differ with respect to how they set the balance between achieving (i) maximal data accessibility - encouraging data submissions by requiring minimal metadata standards, and (ii) maximal data findability, interoperability, and reusability - by requiring much more comprehensive cataloguing of metadata at the risk of discouraging data submissions. A common challenge is the lack of well-defined comprehensive checklists before embarking on sample collections. Efforts to develop these would mean that the appropriate metadata can be captured during the experiment, rather than retrospectively having to determine the key attributes and recover their values from heterogeneous sources. The examples presented above highlight how consistently capturing at least sample provenance can facilitate some retrospective metadata harvesting, but the challenges of doing so remain considerable. Despite general commonalities amongst standards for the whole data lifecycle: data collection, data processing, analysis, annotation, curation, and data deposition, communities recognise that metadata standards are not ‘one-size-fits-all’ because the great variety of research projects means that some degree of flexibility is required. They also recognise the important added value of investing in comprehensive metadata collection. Practically however, the heterogeneity of current solutions limits communities’ abilities to fully exploit the accumulating data to advance biodiversity research.

### Lack of brokering services tailored to communities

Another common challenge across research communities is the lack of comprehensive and dedicated support to help scientists work towards better compliance with FAIR principles. Researchers who are designing and carrying out the sampling and experiments are not necessarily trained with the technical know-how to ensure good data management. In larger consortia there is often more scope for such support, but this has been historically largely responsive rather than being fully integrated from the early planning stages. Funding agencies are increasingly requiring detailed planning on standards for metadata collection, collation, aggregation, dissemination, and archiving, but implementing such plans remains challenging. For individual researchers, this process often constitutes a barrier that prevents their data being made available in the most useful way to the rest of the scientific community. The use cases above highlight some examples of communities that are building brokering services to meet their own needs, but these probably reflect the exception rather than the rule. Brokering services support researchers by maintaining a technical infrastructure for aiding and automating data submission. For example, the Integrated Publishing Toolkit ( IPT 2021) is a free open-source software used to publish and share biodiversity datasets through the GBIF network. Even when such data brokering tools exist for specific communities, users still need support to ensure that they are using the systems correctly: selecting the right standards; employing the right formats; obtaining useful feedback when metadata is not collected properly or missing; and most importantly, a human support mechanism that fits with their domain. The next step, integrating data across different research domains, is often where the greatest metadata loss occurs. If a host resource cannot accommodate certain data types or structures, these remain with the submitter and risk being lost altogether even if provenance is recorded. Proactive communities have recognised many of these challenges and developed brokering tools to meet specific needs, and more generally it is clear that without such supporting services the end-value of the data for biodiversity research is greatly diminished. With the scaling up of production of high-quality sequence data collections and biodiversity research datasets, communities also recognise that ensuring high standards achieved normally through manually curating metadata will not be possible without efficient brokering. This remains practically challenging in many cases as developing and maintaining such dedicated support services to assist researchers with FAIR data brokering is rarely prioritised.

## Recommendations for closer integrations that will shape the future of biodiversity research

Our survey of approaches by which molecular technologies help inform understanding of biodiversity aimed to identify opportunities and priorities to aid strategic thinking. This highlights the emerging critical importance of making use of molecular data to advance understanding of biodiversity in its broadest terms. The four use cases clearly demonstrate that molecular data are now increasingly and routinely used to inform diverse questions on taxonomies, diversity and abundance of microorganisms, the interface with the human food chain, and to increase our understanding of organisms in a wider ecological sense. Also evident is the rapid change in scale, both in terms of foundational whole genomes and derived data, which is creating related challenges across the use cases and more widely in the field. To that end, we therefore make the following recommendations, which we believe are essential for the wider field of biodiversity research to benefit from the vast quantity of molecular data that will be generated in the coming years:

### Biodiversity-related and molecular-focused infrastructures need to collaborate

First and foremost, the key infrastructures in the molecular domain such as ELIXIR, should seek to form strong collaborations with those that span the biodiversity domain, such as (but not limited to) GBIF, DiSSCo, CETAF, ENVRI, CoL, BLR and OBIS. This will be required to meet the challenges associated with the steep scaling up of molecular approaches for the study of biodiversity. Infrastructures should build Communities of Practice that create standards and alignment across the two domains of science. This will support research aimed at discovering, monitoring, characterising, and understanding biodiversity, but also many other areas of research and innovation in the life sciences using genetic diversity as a basis. Infrastructures can benefit from the experience of ELIXIR to independently build solutions to meet specific community needs but maintain interoperability with existing resources. A significant step in this direction will be via a Horizon 2020 funded project to build the Biodiversity Community Integrated Knowledge Library ( BiCIKL). This will bring together a cross-disciplinary set of infrastructures, spanning molecular, taxonomic, literature, museum, and others into a single community focused on addressing biodiversity-related data challenges.

### Taxonomies need to be aligned and harmonised across domains

To address shortcomings in the way taxonomies are handled between the biodiversity and molecular domains we should adopt a common linked data resource. Building on existing resources, taxonomy methodologies need to bridge the gap between identifiers in the molecular domain (e.g. taxID) and taxonomic names in the biodiversity domain, in a manner that is harmonised across repositories. This would provide tools to deal with synonyms and updates, it would enable better understanding of the meaning of a taxonomic name through access to taxonomic treatments, and it would facilitate annotations and links with external data. Harmonisation would also provide researchers with access to a comprehensive and consistent overview of known or accepted taxa names as a proxy of the current state of existing biodiversity to be characterised. This needs to cover all branches of life and be able to accommodate emerging potential species currently only known from sequencing-based studies.

### Metadata needs to be better standardised and universally adopted

To facilitate links across the biodiversity and molecular domains we should develop a consistent set of interoperable metadata standards that are fit-for-purpose and fully integrated into the research lifecycle. This will allow for the connected tracking of accessions, vouchers, and samples with a rich wealth of information captured about their origins (localisation, biome, etc.), and with publications synthesising the emerging knowledge. This has to be associated with a set of technical standards and tools to facilitate data and metadata collection, formatting, and curation, with brokering services to guide the process to completion. Responding to such needs, the ELIXIR Research Data Management Kit ( RDMkit 2021) offers guidance on life sciences data management practices applicable to metadata in biodiversity-related research. Finally, and recognising that standards as described here are only useful if they are widely used, we recommend there is a rigorous drive towards their universal adoption via data brokering and deposition platforms and via publication of results in the scientific literature.

### Approaches for managing molecular data need to be scaled up

As the rate of acquisition grows, and molecular data are increasingly recognised as a common resource with multiple downstream applications, data management solutions need to scale accordingly. It is clear that from barcodes to reference genomes, sequencing hundreds of thousands of species in the near future will generate the foundational data for most biodiversity molecular studies for decades to come. Efficient data management will require national and international investments to build and sustain the required infrastructures. Upscaling the approaches for standardised and common methods for metadata capture, sequence analysis and annotation, as well as curation and archival, is critical if the data are to be re-used as widely as possible at a large scale and across domains. In addition, when operating at this scale, and across many geographies, it is essential now that the core resources are designed to be sustained in the long term.

### Bioinformatics tools and services for biodiversity research need to be prioritised

Continued community-driven development of the analysis tools and services required to take full advantage of the accumulating data should be actively supported. Methods for the analysis of molecular data integrated with biodiversity-related data will continue to evolve and improve, so adopting a fixed approach to data analysis is not a realistic option. Instead, development should proceed in an environment that encourages innovation while building on and connecting to existing tools and services. To achieve this in an efficient manner that benefits the entire community, bioinformatics methods development needs to follow the recommendations on FAIR software ( Katz et al. 2021). To encourage this, we should prioritise the establishment of dedicated recommendations and guidelines for best practices in developing bioinformatics tools and services for biodiversity research. For example, workflows used to analyse biodiversity-related data should be containerised and made easily accessible through BioContainers ( da Veiga Leprevost et al. 2017) or within cloud computing infrastructures. These efforts can benefit from and should build on the ELIXIR tools ecosystem ( ELIXIR 2021b) that aims to help communities find, register and benchmark software tools, while maintaining information standards for these tools, and producing, adopting and promoting best practices for their development.

### Training needs to be widely available to the community and sustained

To encourage and enable the adoption of these recommendations by the end-user communities, we should build common training, capacity building, and outreach activities. This needs to cover all stages of the processes involved, from sampling to data processing and analysis. Training ensures dissemination of the developed tools, resources, and standards to the scientific community and engagement feeds back into refinements and new initiatives to better serve community needs. Sustained support for training connects infrastructure developers like data engineers, service providers, and software developers, with infrastructure users producing and analysing biodiversity-related data. The rewards from prioritising training are evident from the experiences of the ELIXIR Training Platform, through which researchers are empowered with the skills and confidence to use the relevant tools and services and contribute to their continued development.

### The biodiversity community needs to proactively seek common solutions that enable molecular technologies to advance biodiversity research

This survey represents a step in the direction of identifying common challenges and opportunities with respect to how molecular technologies can help inform understanding of biodiversity. The use cases described above show how different research communities are developing initiatives to connect molecular sequence data collections with biodiversity research infrastructures. They represent just a small fraction of ongoing initiatives spanning a wide range of biodiversity studies, some more and others less aware of each other’s activities. Research communities should be proactive in communicating their needs and the solutions to meet them, thereby encouraging cross-community development of tools and resources that multiply benefits and avoid redundancies. The ELIXIR contextualised portfolio of biodiversity informatics resources and services provides a starting point to bringing visibility to existing infrastructures as well as stimulating improved integration. In order to better understand the challenges concerning emerging technologies, scaling up workflows, and ensuring that standards evolve in a coherent manner, we recommend that the community develops a curated, shared, and public understanding of the different types of emerging data, dataflows, repositories, and portals that are necessary to steward up-to-date, comprehensive, complete, and interoperable reference datasets on biodiversity. Such a catalogue of use cases would be a natural output of the Communities of Practice described above in our recommendation on improved collaborations. Through such community-driven initiatives, core sets of standards, approaches, and techniques should be defined that provide all researchers with the means to address critical biodiversity questions by taking advantage of well-connected molecular sequence and biodiversity research infrastructures.

## Data availability

No data are associated with this article.
