# Supplementary material for: Recommendations for connecting molecular sequence and biodiversity research infrastructures through ELIXIR
Source: F1000Res. 2022 Aug 1;10:ELIXIR-1238. Originally published 2021 Dec 3. [Version 2] doi: 10.12688/f1000research.73825.2 (PMC9360911; doi:10.12688/f1000research.73825.2)
Supplement: Peer review history

# Peer review: Reviewer response for version 2

**DOI:** 10.5256/f1000research.136321.r146169

**Published:** 2022-08-08

**Authors:** Anders Andersson

**Affiliations:** 1 Department of Gene Technology, School of Engineering Sciences in Chemistry, Biotechnology and Health, KTH Royal Institute of Technology, Science for Life Laboratory, Stockholm, Sweden

The authors have addressed the concerns raised and I'm happy with the revised version of the article.

Is the topic of the opinion article discussed accurately in the context of the current literature?

Yes

Are arguments sufficiently supported by evidence from the published literature?

Yes

Are all factual statements correct and adequately supported by citations?

Yes

Are the conclusions drawn balanced and justified on the basis of the presented arguments?

Yes

Reviewer Expertise:

Microbial ecology and evolution. Bioinformatics.

I confirm that I have read this submission and believe that I have an appropriate level of expertise to confirm that it is of an acceptable scientific standard.

# Peer review: Reviewer response for version 1

**DOI:** 10.5256/f1000research.77505.r119683

**Published:** 2022-03-31

**Authors:** Anders Andersson

**Affiliations:** 1 Department of Gene Technology, School of Engineering Sciences in Chemistry, Biotechnology and Health, KTH Royal Institute of Technology, Science for Life Laboratory, Stockholm, Sweden

The opinion article by Waterhouse et al. "Recommendations for connecting molecular sequence and biodiversity research infrastructures through ELIXIR" goes through a number of use cases on how biodiversity information from DNA sequencing data can be integrated into biodiversity platforms and also highlights challenges related to this. Towards the end, the authors, as the title promises, provide some recommendations on what type of infrastructure initiatives should be funded, to help the biodiversity field overcome the challenges.

 This paper has many good points and the authors have pinpointed several of the caveats of integrating molecular data with biodiversity platforms. And it also provides some concrete advice on projects/initiatives to overcome the obstacles. But the paper is extremely long - 28 pages - and sometimes difficult to read. For example, it took me five attempts to grasp the meaning of this sentence: “Our survey of approaches by which molecular technologies help inform understanding of biodiversity aimed to identify opportunities and priorities to aid strategic thinking”. And the following sentence doesn't provide much aid: "This highlights the emerging critical importance of making use of molecular data to advance understanding of biodiversity in its broadest terms.”. I would thus recommend the authors simplify the text a bit. Likewise, I think the paper would benefit from being shortened, otherwise, there is a risk many readers will never reach the recommendations at the end (which, given the title, is probably the main point of this opinion paper).

 Here some specific suggestions (page numbers refer to the pdf version of the article):

 Abstract: "To identify opportunities, highlight priorities, and aid strategic thinking, here we survey approaches by which molecular technologies help inform understanding of biodiversity." -> (I suggest) "Here we survey approaches by which molecular technologies help inform understanding of biodiversity, in order to identify opportunities, highlight priorities, and aid strategic thinking."

 Abstract: “Increasing knowledge of marine biodiversity” -> “increasing knowledge of marine biodiversity”

 p. 3: “at genetic, species, and ecosystem” -> “at population, community and ecosystem levels” (all those levels involve genetics)

 p. 3: “millions of years of evolution” -> “billions of years of evolution” (life on Earth arose 3-4 billion years ago)

 p. 4: "These examples help to formulate more formal definitions: (i) molecular sequence data collection initiatives are producing and collating reference catalogues of genetic and genomic biodiversity on Earth; and (ii) biodiversity research infrastructures are capturing knowledge from scientific collections, observations, and the literature, and building resources of biodiversity information for all Earth’s organisms. Here we identify opportunities to connect these."

 - I find the definition (i) incomplete: in addition to producing reference catalogues (e.g. genomes or marker genes) they, importantly, also contain sequencing datasets from the field that hold information on species occurrences (and sometimes intra-specific diversity) in samples (i.e. metagenomic and metabarcoding datasets). The description of MGnify on page 7 illustrates this. Maybe this is what is meant by "collating" but that was not clear to me.

 Table 1: Add GTDB (https://gtdb.ecogenomic.org/)

 p. 6: Add a brief description of GTDB, for example to the first paragraph of page 6. GTDB is rapidly establishing itself as the standard for cataloguing prokaryotic diversity and a good example of how (meta)genomics can aid in improving taxonomies.

 p. 8: "In this context, while seeking a new experimental design for molecular characterisation of specific organisms, the absence of unique identifiers (i.e. taxIDs) represents an important issue in collecting the most comprehensive information related to the organisms of interest."

 - This sentence has unclear meaning to me, what is meant by "new experimental design" here?

 p. 18: “These efforts can benefit from and should build on the ELIXIR tools ecosystem (ELIXIR 2021b) that aims to help communities find, register and benchmark software tools, while maintaining information standards for these tools, and producing, adopting and promoting best practices for their development.”.

 - I'm not really in favour of using the term "should" here. There are other examples of software tool collaborations that fulfil the FAIR requirements such as the nf-core collaboration (https://nf-co.re/) with pipelines/tools used by thousands of researchers and many sequencing facilities.

Is the topic of the opinion article discussed accurately in the context of the current literature?

Yes

Are arguments sufficiently supported by evidence from the published literature?

Yes

Are all factual statements correct and adequately supported by citations?

Yes

Are the conclusions drawn balanced and justified on the basis of the presented arguments?

Yes

Reviewer Expertise:

Microbial ecology and evolution. Bioinformatics.

I confirm that I have read this submission and believe that I have an appropriate level of expertise to confirm that it is of an acceptable scientific standard, however I have significant reservations, as outlined above.

# Response

**Published:** 2022-07-18

**Authors:** Jerry Lanfear

**Affiliations:** ELIXIR, UK

The opinion article by Waterhouse et al. "Recommendations for connecting molecular sequence and biodiversity research infrastructures through ELIXIR" goes through a number of use cases on how biodiversity information from DNA sequencing data can be integrated into biodiversity platforms and also highlights challenges related to this. Towards the end, the authors, as the title promises, provide some recommendations on what type of infrastructure initiatives should be funded, to help the biodiversity field overcome the challenges.

 This paper has many good points and the authors have pinpointed several of the caveats of integrating molecular data with biodiversity platforms. And it also provides some concrete advice on projects/initiatives to overcome the obstacles. But the paper is extremely long - 28 pages - and sometimes difficult to read. For example, it took me five attempts to grasp the meaning of this sentence: “Our survey of approaches by which molecular technologies help inform understanding of biodiversity aimed to identify opportunities and priorities to aid strategic thinking”. And the following sentence doesn't provide much aid: "This highlights the emerging critical importance of making use of molecular data to advance understanding of biodiversity in its broadest terms.”. I would thus recommend the authors simplify the text a bit. Likewise, I think the paper would benefit from being shortened, otherwise, there is a risk many readers will never reach the recommendations at the end (which, given the title, is probably the main point of this opinion paper).

**Response: We thank the reviewer for noting the positive points and for the constructive criticisms with respect to addressing readability issues, also noted by reviewer 1. We have made simplifications and rephrased complex statements to more clearly convey the main messages. Regarding the length, we recognise that describing the four use cases in the manuscript adds substantially to the overall content, but we believe these details are necessary as they provide the basis from which to develop meaningful recommendations. We agree that the recommendations are the main point of the paper, and we believe that many readers will be inclined to focus on this section along with one or two of the use cases most closely aligned with their own research fields. Thus, while attempting to be more concise throughout the manuscript we would prefer not to dramatically shorten or discard any particular section. We have also rephrased the two sentences highlighted here to improve readability.**

 Here some specific suggestions (page numbers refer to the pdf version of the article):

 Abstract: "To identify opportunities, highlight priorities, and aid strategic thinking, here we survey approaches by which molecular technologies help inform understanding of biodiversity." -> (I suggest) "Here we survey approaches by which molecular technologies help inform understanding of biodiversity, in order to identify opportunities, highlight priorities, and aid strategic thinking."

**Response: Agree, updated.**

 Abstract: “Increasing knowledge of marine biodiversity” -> “increasing knowledge of marine biodiversity”

**Response: fixed**

 p. 3: “at genetic, species, and ecosystem” -> “at population, community and ecosystem levels” (all those levels involve genetics)

 Response: Agree that the proposed formulation is better, updated.

 p. 3: “millions of years of evolution” -> “billions of years of evolution” (life on Earth arose 3-4 billion years ago)

**Response: Agree, updated.**

 p. 4: "These examples help to formulate more formal definitions: (i) molecular sequence data collection initiatives are producing and collating reference catalogues of genetic and genomic biodiversity on Earth; and (ii) biodiversity research infrastructures are capturing knowledge from scientific collections, observations, and the literature, and building resources of biodiversity information for all Earth’s organisms. Here we identify opportunities to connect these."

 - I find the definition (i) incomplete: in addition to producing reference catalogues (e.g. genomes or marker genes) they, importantly, also contain sequencing datasets from the field that hold information on species occurrences (and sometimes intra-specific diversity) in samples (i.e. metagenomic and metabarcoding datasets). The description of MGnify on page 7 illustrates this. Maybe this is what is meant by "collating" but that was not clear to me.

**Response: We agree that the definition should be more clearly broadened to encompass other sequencing datasets and have updated the text accordingly.**

 Table 1: Add GTDB (https://gtdb.ecogenomic.org/)

**Response: The examples provided in Table 1 are focused on international projects and umbrella initiatives producing (meta) genomes, (meta) transcriptomes, and/or DNA barcodes. GTDB seems to fit more the profile of a consumer of such data and therefore we do not think it represents an example of the type of project we wish to highlight here.**

 p. 6: Add a brief description of GTDB, for example to the first paragraph of page 6. GTDB is rapidly establishing itself as the standard for cataloguing prokaryotic diversity and a good example of how (meta)genomics can aid in improving taxonomies.

**Response: We agree that this is a good example and we have added GTDB in the discussion of microbe-focused sequencing initiatives.**

 p. 8: "In this context, while seeking a new experimental design for molecular characterisation of specific organisms, the absence of unique identifiers (i.e. taxIDs) represents an important issue in collecting the most comprehensive information related to the organisms of interest."

 - This sentence has unclear meaning to me, what is meant by "new experimental design" here?

**Response: Indeed, ‘new experimental design’ could be misleading, we have rephrased to improve clarity**

 p. 18: “These efforts can benefit from and should build on the ELIXIR tools ecosystem (ELIXIR 2021b) that aims to help communities find, register and benchmark software tools, while maintaining information standards for these tools, and producing, adopting and promoting best practices for their development.”.

 - I'm not really in favour of using the term "should" here. There are other examples of software tool collaborations that fulfil the FAIR requirements such as the nf-core collaboration (https://nf-co.re/) with pipelines/tools used by thousands of researchers and many sequencing facilities.

**Response: Indeed the use of “should” here was meant to echo the sentiment above relating to “building on and connecting to existing tools and services” but as it could be misinterpreted we have reworded to remove ambiguity.**

# Peer review: Reviewer response for version 1

**DOI:** 10.5256/f1000research.77505.r115285

**Published:** 2022-01-05

**Authors:** Donald Hobern

**Affiliations:** 1 Species 2000, Leiden, The Netherlands; 2 International Barcode of Life, Guelph, Canada; 3 Atlas of Living Australia, Canberra, Australia; 4 Australian Plant Phenomics Facility, Adelaide, Australia

This paper addresses probably the most significant opportunity for data-driven innovation and transformation in taxonomy, biogeography, ecology, conservation and biosecurity, with major implications for sustainability and food security.

 The paper is well structured and clearly demonstrates the potential and challenges. I've divided my comments as follows: 1) clarifications of detail (minor) in regard to some of the referenced initiatives, 2) a few major initiatives that are highly relevant but not referenced, 3) suggestions to make the text clearer and more readable.

 I use page numbers from the PDF version downloadable on 4 January 2022.

**Clarifications of detail**

 Page 4: The preferred abbreviation for Catalogue of Life is now (since 2021) fully capitalised (COL).

 Page 4: "The taxonomic frameworks are built ..." - this seems to refer specifically to GBIF's taxonomic framework, so perhaps replace "The" with "Its". More importantly, by far the largest contribution to GBIF's framework is COL (see https://data-blog.gbif.org/post/gbif-backbone-taxonomy/) - this provides the major structure and other published resources (including BLR) augment it.

 Page 6 - "The main reference libraries include ..." - BOLD is the main reference library that supports iBOL. iBOL is not a separate library. I recommend rewriting "and the International Barcode of Life" as "maintained by the International Barcode of Life".

 Page 6 - "(e.g. NCBI or GBIF)" - as noted above, COL is the core of the GBIF backbone and is used in many other contexts. It may be better to reference "(NCBI, COL or GBIF)".

 Page 7 - "For example, connecting GBIF with the UNITE database" - Note that GBIF has integrated both BOLD and UNITE in this way - both now contribute molecular OTUs (in BOLD's case BINs) that appear as part of the GBIF taxonomic framework.

 Page 7 - "Reciprocally, traditional biodiversity data and resources can help inform" - while this is true, it is not clear how the authors expect the benefits to be developed in this direction. Navigation from molecular data to a corpus of knowledge about associated taxa is *relatively* simple to achieve, but the published literature is insufficiently structured or parsed to support meaningful inference to support machine-driven analysis of genetic and genomic data.

 Page 8 - "The NCBI taxonomy database" - The listed number of synonyms is important, but should be put in context of the current version of COL including 1.95 million accepted species names and another 2 million synonyms.

 Page 10 - "An additional source of information on taxon names" - It is important to highlight the scale and diversity of information resources and datasets relating to biodivesity, but these are so heterogeneous that it is unhelpful to treat them monolithically. Lumping them together leaves taxonomic identifiers as the only possible connection point. In practice, the field of biodiversity informatics needs to digest these resources into digital objects that fall into more precise classes (specimen, ecosystem, species, gene, sampling event, trait, etc.).

 Page 13 - Figure 3 caption - "They can also archive their datasets at GBIF without any clearance." - Unless I am missing something, this would be better expressed as "publish their datasets to GBIF" since GBIF does not currently assume responsibility for archival of data published to the network (although such archival often de facto occurs).

 Page 16 - "Missing and non-matched taxIDs give an incomplete and inconsistent view" - All that is written here is true, but an associated and often neglected issue is the uncertainty associated with taxonomic identifications. A name may be correctly interpreted according to a perfect taxonomic framework, while all the time being based on misidentification. This aspect overlays everything written here and needs to be acknowledged. Of course, this is also a key area in which the fusion of genetic/genomic and other data can bring big benefits. Ideally taxonomic type specimens will end up serving as anchor points not only for morphological descriptions but also as DNA vouchers that can be used to label the corresponding molecular OTUs and validate field-collected data.

 Page 17 - "Efforts to develop these would mean that the appropriate metadata can be captured during the experiment" - True, but it is important that we distinguish clearly within the metadata between elements co-collected with the sample of interest and elements added subsequently via interpolation, look-up, etc.

 Page 17 - "Even when such data brokering tools exist for specific communities" - Another source of difficulty is inconsistent rigor in defining or interpreting even widely adopted standards. Mapping data from different studies will involve compromises and ambiguities that may not be apparent either to those sharing the data or to consumers of the data.

 Page 18 - "Metadata needs to be better standardised and universally adopted" - It may be worthwhile to clarify the scope of what is intended by "metadata" - FAIR data standards should include consideration of data structures and packaging models to ensure that users can correctly find and interpret all elements. This is more complex than adopting vocabulaties, etc.

 Page 18 - "Bioinformatics tools and services for biodiversity research need to be prioritised" - It may be worthwhile to acknowledge that we do not need monolithic solutions here. We need minimum information standards, stable identifiers and provenance information, good generalisable packaging mechanisms and a software ecosystem that assists with point-to-point or data-class to data-class transformations. Satellite imagery may be a good analogy. Downstream consumers need well-referenced products such as NDVI - these become the components of interest for other more targeted applications. In the same way, we may be best off focusing on a modular approach - develop robust taxonomic frameworks and associated tools, map molecular hypotheses against these frameworks, ensure that data from samples can be consumed as Darwin Core Occurrences and Events, etc.

**Other initiatives**

 Page 7 - "Ongoing efforts to coordinate traditional biodiversity infrastructures" - As well as the initiatives referenced in this paragraph, GBIF and partners have organised two global conferences, each leading to a publication focused on building such coordination (Hobern *et al.* 2012, Hobern *et al.* 2019). The later event led to the call for an alliance for biodiversity knowledge ( https://www.biodiversityinformatics.org/) which is highly relevant to this paper as an umbrella for cross-infrastructure collaborations. The alliance is also applicable to page 18 "Biodiversity-related and molecular-focused infrastructures need to collaborate".

 Page 8 - Use case 1, paragraph 1 - A topical collection of six papers has recently been published in Organisms Diversity and Evolution from work carried out under the auspices of IUBS. This collection specifically explores the need for a shared taxonomic framework and makes proposals for the required collaboration (Towards a global list of accepted species, see here). Citations for all six papers provided. This is especially applicable to page 18 "Taxonomies need to be aligned and harmonised across domains".

**Readability**

 It may be a relatively minor issue, but many sentences throughout the document are unnecessarily hard to read because the central ideas are delayed to the end of the sentence and/or a passive voice is unnecessarily used. For example, in the abstract, consider rewriting "As a research infrastructure developing services and technical solutions that help integrate and coordinate life science resources across Europe, ELIXIR is a key player" as "ELIXIR plays a key role as a research infrastructure that develops services and technical solutions that help integrate and coordinate life science resources across Europe". I found I needed to re-read several passages a few times to get their sense. In almost all cases, the concepts were correct and important, but obscured by word order.

 Page 8 - use case 1 seems in particular need of a rewrite to improve clarity. The first sentence, ("Creating a comprehensive taxonomy linked ...") does not make sense and certainly needs to be rewritten. 

 Page 11 - "The MAR database entries are cross-referenced with ENA and the World Register of Marine Species (WoRMS) (Vandepitte et al. 2018) records" - no need for the word "records".

 Page 11 - "On the one hand, the large and growing variety of observations taken during oceanic sampling (Gorsky et al. 2019) have posed many data management challenges." - "has posed".

 Page 13 - "Long-standing scientific interests" - "interest".

 Page 15 - "This species, experiment and sample metadata" - may be clearer as "This metadata on species, experimennts and samples".

 Page 18 - "It is clear that from barcodes to reference genomes, sequencing hundreds of thousands of species in the near future will generate the foundational data for most biodiversity molecular studies for decades to come." - this sentence is awkward and could be rewritten.

Is the topic of the opinion article discussed accurately in the context of the current literature?

Yes

Are arguments sufficiently supported by evidence from the published literature?

Yes

Are all factual statements correct and adequately supported by citations?

Yes

Are the conclusions drawn balanced and justified on the basis of the presented arguments?

Yes

Reviewer Expertise:

Biodiversity informatics including management of taxonomic and DNA barcode data, use of data in taxonomy, ecology and agriculture.

I confirm that I have read this submission and believe that I have an appropriate level of expertise to confirm that it is of an acceptable scientific standard.

## References

[1] : Global Biodiversity Informatics Outlook: Delivering biodiversity knowledge in the information age. *Global Biodiversity Information Facility* .2012; https://doi.org/10.15468/6JXA-YB44 10.15468/6JXA-YB44

[2] : Connecting data and expertise: a new alliance for biodiversity knowledge. *Biodivers Data J* .2019;7: 10.3897/BDJ.7.e33679 e33679 10.3897/BDJ.7.e33679 30886531PMC6420472

[3] : Towards a global list of accepted species I. Why taxonomists sometimes disagree, and why this matters. *Organisms Diversity & Evolution* .2021;21(4) : 10.1007/s13127-021-00495-y 615-622 10.1007/s13127-021-00495-y

[4] : Towards a global list of accepted species II. Consequences of inadequate taxonomic list governance. *Organisms Diversity & Evolution* .2021;21(4) : 10.1007/s13127-021-00518-8 623-630 10.1007/s13127-021-00518-8

[5] : Towards a global list of accepted species III. Independence and stakeholder inclusion. *Organisms Diversity & Evolution* .2021;21(4) : 10.1007/s13127-021-00496-x 631-643 10.1007/s13127-021-00496-x

[6] : Towards a global list of accepted species IV: Overcoming fragmentation in the governance of taxonomic lists. *Organisms Diversity & Evolution* .2021;21(4) : 10.1007/s13127-021-00499-8 645-655 10.1007/s13127-021-00499-8

[7] : Towards a global list of accepted species V. The devil is in the detail. *Organisms Diversity & Evolution* .2021;21(4) : 10.1007/s13127-021-00504-0 657-675 10.1007/s13127-021-00504-0

[8] : Towards a global list of accepted species VI: The Catalogue of Life checklist. *Organisms Diversity & Evolution* .2021;21(4) : 10.1007/s13127-021-00516-w 677-690 10.1007/s13127-021-00516-w

# Response

**Published:** 2022-07-18

**Authors:** Jerry Lanfear

**Affiliations:** ELIXIR, UK

This paper addresses probably the most significant opportunity for data-driven innovation and transformation in taxonomy, biogeography, ecology, conservation and biosecurity, with major implications for sustainability and food security.

 The paper is well structured and clearly demonstrates the potential and challenges. I've divided my comments as follows: 1) clarifications of detail (minor) in regard to some of the referenced initiatives, 2) a few major initiatives that are highly relevant but not referenced, 3) suggestions to make the text clearer and more readable.

 I use page numbers from the PDF version downloadable on 4 January 2022.

**Response: We thank the reviewer for recognising the importance of this Opinion Piece and especially for the detailed constructive feedback that has undoubtedly helped to improve the manuscript substantially.**

 Clarifications of detail

 Page 4: The preferred abbreviation for Catalogue of Life is now (since 2021) fully capitalised (COL).

**Response: Updated to COL throughout, including in Figure 1.**

 Page 4: "The taxonomic frameworks are built ..." - this seems to refer specifically to GBIF's taxonomic framework, so perhaps replace "The" with "Its". More importantly, by far the largest contribution to GBIF's framework is COL (see https://data-blog.gbif.org/post/gbif-backbone-taxonomy/) - this provides the major structure and other published resources (including BLR) augment it.

**Response: Updated accordingly.**

 Page 6 - "The main reference libraries include ..." - BOLD is the main reference library that supports iBOL. iBOL is not a separate library. I recommend rewriting "and the International Barcode of Life" as "maintained by the International Barcode of Life".

**Response: Updated accordingly.**

 Page 6 - "(e.g. NCBI or GBIF)" - as noted above, COL is the core of the GBIF backbone and is used in many other contexts. It may be better to reference "(NCBI, COL or GBIF)".

**Response: Updated accordingly.**

 Page 7 - "For example, connecting GBIF with the UNITE database" - Note that GBIF has integrated both BOLD and UNITE in this way - both now contribute molecular OTUs (in BOLD's case BINs) that appear as part of the GBIF taxonomic framework.

**Response: Our second example in this section (Canadian invertebrate fauna) presents a specific case of how BOLD integrates with GBIF, so although somewhat implicit this integration is already noted and we prefer to leave it as it is rather than expand this section.**

 Page 7 - "Reciprocally, traditional biodiversity data and resources can help inform" - while this is true, it is not clear how the authors expect the benefits to be developed in this direction. Navigation from molecular data to a corpus of knowledge about associated taxa is *relatively* simple to achieve, but the published literature is insufficiently structured or parsed to support meaningful inference to support machine-driven analysis of genetic and genomic data.

**Response: We agree that currently there are many challenges that still need to be overcome to achieve this and have edited the text to reflect this as a future goal rather than a current reality.**

 Page 8 - "The NCBI taxonomy database" - The listed number of synonyms is important, but should be put in context of the current version of COL including 1.95 million accepted species names and another 2 million synonyms.

**Response: For context we have added this after the reference to Figure 1.**

 Page 10 - "An additional source of information on taxon names" - It is important to highlight the scale and diversity of information resources and datasets relating to biodivesity, but these are so heterogeneous that it is unhelpful to treat them monolithically. Lumping them together leaves taxonomic identifiers as the only possible connection point. In practice, the field of biodiversity informatics needs to digest these resources into digital objects that fall into more precise classes (specimen, ecosystem, species, gene, sampling event, trait, etc.).

**Response: Although this case study is focused on taxonomy we agree that highlighting the role of digital objects is important and have updated the text accordingly.**

 Page 13 - Figure 3 caption - "They can also archive their datasets at GBIF without any clearance." - Unless I am missing something, this would be better expressed as "publish their datasets to GBIF" since GBIF does not currently assume responsibility for archival of data published to the network (although such archival often de facto occurs).

**Response: Updated accordingly.**

 Page 16 - "Missing and non-matched taxIDs give an incomplete and inconsistent view" - All that is written here is true, but an associated and often neglected issue is the uncertainty associated with taxonomic identifications. A name may be correctly interpreted according to a perfect taxonomic framework, while all the time being based on misidentification. This aspect overlays everything written here and needs to be acknowledged. Of course, this is also a key area in which the fusion of genetic/genomic and other data can bring big benefits. Ideally taxonomic type specimens will end up serving as anchor points not only for morphological descriptions but also as DNA vouchers that can be used to label the corresponding molecular OTUs and validate field-collected data.

**Response: This is indeed a very important point that we did not address specifically, we have added “Taxon misidentifications” to this section to highlight this while not going into details to try to keep this section on common challenges concise, instead adding a sentence to the use case 1 section earlier to elaborate this point.**

 Page 17 - "Efforts to develop these would mean that the appropriate metadata can be captured during the experiment" - True, but it is important that we distinguish clearly within the metadata between elements co-collected with the sample of interest and elements added subsequently via interpolation, look-up, etc.

**Response: We agree with this distinction and have updated the text to include this important point.**

 Page 17 - "Even when such data brokering tools exist for specific communities" - Another source of difficulty is inconsistent rigor in defining or interpreting even widely adopted standards. Mapping data from different studies will involve compromises and ambiguities that may not be apparent either to those sharing the data or to consumers of the data.

**Response: We agree that even within domains (between studies) there are ambiguities and have updated the text to specifically mention this before considering cross-domain issues.**

 Page 18 - "Metadata needs to be better standardised and universally adopted" - It may be worthwhile to clarify the scope of what is intended by "metadata" - FAIR data standards should include consideration of data structures and packaging models to ensure that users can correctly find and interpret all elements. This is more complex than adopting vocabulaties, etc.

**Response: We agree that data structures and packaging models are also important for FAIR. However, here we focus on metadata solely as an information collection, standardisation and curation mechanism, as this is a vital first step in how knowledge is represented within a biodiversity project, i.e. sample collection metadata as defined in the Darwin Tree of Life. Data structures and packaging models are primarily concerned with the consumption of biodiversity data, and if the metadata provided at the outset is high quality and standardised, downstream tools and APIs can be varied yet still remain FAIR. We modified the text to clarify the focus here on the first steps in the process of metadata collection.**

 Page 18 - "Bioinformatics tools and services for biodiversity research need to be prioritised" - It may be worthwhile to acknowledge that we do not need monolithic solutions here. We need minimum information standards, stable identifiers and provenance information, good generalisable packaging mechanisms and a software ecosystem that assists with point-to-point or data-class to data-class transformations. Satellite imagery may be a good analogy. Downstream consumers need well-referenced products such as NDVI - these become the components of interest for other more targeted applications. In the same way, we may be best off focusing on a modular approach - develop robust taxonomic frameworks and associated tools, map molecular hypotheses against these frameworks, ensure that data from samples can be consumed as Darwin Core Occurrences and Events, etc.

**Response: We had hoped to have conveyed this with phrases such as “adopting a fixed approach to data analysis is not a realistic option” and “development should proceed in an environment that encourages innovation while building on and connecting to existing tools and services”. Describing these concepts with the suggested term “modular approach” works well to reinforce these ideas, so we have taken this on board and updated the paragraph to more clearly reflect this message.**

 Other initiatives

 Page 7 - "Ongoing efforts to coordinate traditional biodiversity infrastructures" - As well as the initiatives referenced in this paragraph, GBIF and partners have organised two global conferences, each leading to a publication focused on building such coordination (Hobern et al. 2012, Hobern et al. 2019). The later event led to the call for an alliance for biodiversity knowledge (https://www.biodiversityinformatics.org/) which is highly relevant to this paper as an umbrella for cross-infrastructure collaborations. The alliance is also applicable to page 18 "Biodiversity-related and molecular-focused infrastructures need to collaborate".

**Response: These are indeed important syntheses of efforts to build such coordination, we have updated both paragraphs to highlight the relevance of the alliance for cross-infrastructure collaborations.**

 Page 8 - Use case 1, paragraph 1 - A topical collection of six papers has recently been published in Organisms Diversity and Evolution from work carried out under the auspices of IUBS. This collection specifically explores the need for a shared taxonomic framework and makes proposals for the required collaboration (Towards a global list of accepted species, see here). Citations for all six papers provided. This is especially applicable to page 18 "Taxonomies need to be aligned and harmonised across domains".

**Response: We agree that this topical collection exemplifies many of the issues faced in this domain and have now specifically mentioned this in the text.**

 Readability

 It may be a relatively minor issue, but many sentences throughout the document are unnecessarily hard to read because the central ideas are delayed to the end of the sentence and/or a passive voice is unnecessarily used. For example, in the abstract, consider rewriting "As a research infrastructure developing services and technical solutions that help integrate and coordinate life science resources across Europe, ELIXIR is a key player" as "ELIXIR plays a key role as a research infrastructure that develops services and technical solutions that help integrate and coordinate life science resources across Europe". I found I needed to re-read several passages a few times to get their sense. In almost all cases, the concepts were correct and important, but obscured by word order.

**Response: We have been through the text and specifically identified sentences that would benefit from rearrangements as suggested to improve readability.**

 Page 8 - use case 1 seems in particular need of a rewrite to improve clarity. The first sentence, ("Creating a comprehensive taxonomy linked ...") does not make sense and certainly needs to be rewritten.

**Response: There are two aspects in a single authoritative list. Going forwards a single list seems obvious, as proposed by Garnett and colleagues the way to go. However, all the legacy data requires a list to include all the synonyms, misidentifications, spelling variants and in order to decide, access to the respective taxonomic treatments. To clarify we use the language used by Garnett et al. that introduces the set of six IUBS commissioned papers mentioned above.**

 Page 11 - "The MAR database entries are cross-referenced with ENA and the World Register of Marine Species (WoRMS) (Vandepitte et al. 2018) records" - no need for the word "records".

**Response: Fixed.**

 Page 11 - "On the one hand, the large and growing variety of observations taken during oceanic sampling (Gorsky et al. 2019) have posed many data management challenges." - "has posed".

**Response: Fixed.**

 Page 13 - "Long-standing scientific interests" - "interest".

**Response: Fixed.**

 Page 15 - "This species, experiment and sample metadata" - may be clearer as "This metadata on species, experimennts and samples".

**Response: Agree, updated.**

 Page 18 - "It is clear that from barcodes to reference genomes, sequencing hundreds of thousands of species in the near future will generate the foundational data for most biodiversity molecular studies for decades to come." - this sentence is awkward and could be rewritten.

**Response: Agreed, we have re-worked this sentence for clarity.**
